# Hsp90 is important for fecundity, longevity, and buffering of cryptic deleterious variation in wild fly populations

**DOI:** 10.1186/1471-2148-12-25

**Published:** 2012-02-27

**Authors:** Bing Chen, Andreas Wagner

**Affiliations:** 1 Institute of Zoology, Chinese Academy of Sciences, 100101 Beijing, China; 2 Institute of Evolutionary Biology and Environmental Studies, University of Zurich, 8057 Zurich, Switzerland; 3 The Swiss Institute of Bioinformatics, Quartier Sorge-Batiment Genopode, 1015 Lausanne, Switzerland; 4 The Santa Fe Institute, 1399 Hyde Park Road, Santa Fe, New Mexico 87501, USA

## Abstract

**Background:**

In the laboratory, the *Drosophila melanogaster *heat shock protein Hsp90 can buffer the phenotypic effects of genetic variation. Laboratory experiments either manipulate Hsp90 activity pharmacologically, or they induce mutations with strong effects in the gene *Hsp83*, the single-copy fly gene encoding Hsp90. It is unknown whether observations from such laboratory experiments are relevant in the wild.

**Results:**

We here study naturally occurring mutations in *Hsp83*, and their effects on fitness and phenotypic buffering in flies derived from wild populations. We examined more than 4500 flies from 42 *Drosophila *populations distributed world-wide for insertions or deletions of mobile DNA in or near the *Hsp83 *gene. The insertions we observed occur at low population frequencies, and reduce *Hsp83 *gene expression. In competition experiments, mutant flies performed much more poorly than wild-type flies. Mutant flies were also significantly less fecund and shorter-lived than wild-type flies, as well as less well buffered against cryptic deleterious variation, as we show through inbreeding experiments. Specifically, in *Hsp83 *mutant flies female fecundity dropped to much lower levels after inbreeding than in wild-type flies. At even slightly elevated temperatures, inbred mutant *Hsp83 *populations went extinct, whereas inbred wild-type populations persisted.

**Conclusions:**

Our work shows that Hsp90, a regulator of the stress response and of signaling, helps buffer deleterious variation in fruit flies derived from wild population, and that its buffering role becomes even more important under heat stress.

## Background

How does genotypic variation affect phenotypic variation? And how might genes modulate the relationship between genotype and phenotype? These are central questions in evolutionary biology. In recent years, it has become clear that some genes play a special role in this relationship. These genes encode chaperones, proteins that assist other proteins in folding, and that can help refold misfolded proteins [1-3]. Protein misfolding can result from mutations in protein coding regions [3,4]. It can also result from environmental changes, such as heat stress, which can lead to protein denaturation [5]. Because proteins are involved in forming and maintaining every phenotypic trait, misfolded proteins often have detrimental effects on phenotypes [2,3]. Proteins that can mitigate these effects can render organisms more robust against genetic or environmental perturbations. Thus, chaperones are one of several ways in which phenotypes can become robust to genetic and environmental change [6]. Robustness to genetic and environmental change are often associated with one another [7-11], although exceptions may exist [12]. On evolutionary time scales, robustness to genetic change has an important consequence on the genetic constitution of a population: It allows mutations to accumulate that are not phenotypically visible, precisely because phenotypes are robust to such mutations. The resulting genetic variation is often also called cryptic variation [13,14]. Such variation need not stay cryptic forever, however. It can become phenotypically visible in the presence of yet other mutations or after environmental change [7,14-16]. The resulting phenotypic change can be detrimental, but also beneficial, leading to new evolutionary adaptations [8,10,15,17]. The earliest hints that cryptic variation exists, and that it can be transformed into new phenotypes came from experiments by Waddington which induced new phenotypes through environmental change, and which demonstrated that the induced phenotypic changes can have a genetic basis [18]. In the recent past, many further studies have demonstrated the existence of cryptic variation, and its potential phenotypic consequences. These studies focused on a broad range of phenotypes, from molecular phenotypes to macroscopic traits of multicellular organisms [16,19-22].

Because chaperones help confer robustness, their activity is also a prominent cause of cryptic variation [3,8,23]. An especially important chaperone is the heat shock protein Hsp90, which occurs in many organisms that range from microbes to humans [1,20,24,25]. What makes Hsp90 unique is that its "client" proteins, proteins whose integrity it helps maintain, are extremely diverse, and that they are involved in cell communication and signaling processes [1,5]. Such processes are especially important while a complex organism with many macroscopic traits develops from a single cell. In other words, Hsp90 affects development and thus macroscopic phenotypes. For example, impairing Hsp90 function through engineered mutations or pharmacological treatment-both in the laboratory-can increase phenotypic diversity in organisms as different as *Drosophila*, Arabidopsis, and zebrafish [7,16,26]. This phenotypic diversity is a reflection of genotypic diversity, that is, of variation that was cryptic before Hsp90 function was impaired [24].

With some exceptions [8,15,27], most experiments that studied Hsp90's effects on fitness and variation buffering focus on laboratory strains [24,28-30]. In addition, they use strong gain-of-function or loss-of-function mutations with dramatic, often lethal phenotypic effects [31,32]. Such mutations can usually not be maintained as homozygotes, and would rarely if ever be tolerated in a wild population [16,32,33]. Even disruption of Hsp90 expression through RNA interference may have large effects on phenotypes [15]. Other ways of manipulating Hsp90 activity include the use of pharmaceuticals known to interact with Hsp90, such as geldanamycin [7,8,16], which wild populations may also rarely encounter.

In contrast to such strong, artificial manipulations in the laboratory, one might expect to encounter mutations with milder effects in the wild, such as regulatory mutations that affect gene expression. In the fruit fly *Drosophila*, a specific class of such regulatory mutations are indeed well known to affect the expression of heat shock genes [34]. These mutations are insertions of a transposable element, the *P*-element, into the regulatory region of heat shock genes. Such mutations have been characterized for a variety of heat shock genes, including *Hsp83, Hsp70, Hsp67Ba, Hsp27, Hsp26, Hsp23, Hsp22*, and *Hsrω *[34,35]. They affect gene expression, thermotolerance [35-37], development [35] and longevity [35]. The likely reason why *P *elements often insert into these genes' regulatory regions is their high expression level, which requires a de-condensed and nucleosome-free chromatin conformation near the gene [38,39]. Such an "open" chromatin conformation makes a gene susceptible to insertion by transposable elements [34].

In the fruit fly *Drosophila*, where the role of Hsp90 in variation buffering was first discovered [16], Hsp90 is encoded by the single copy essential gene *Hsp83*. This gene is expressed at higher levels during normal development than some other heat shock proteins, including *Hsp22, Hsp23, Hsp26, Hsp27*, and *Hsp70 *[40,41]. Hsp90 is expressed constitutively, and at higher levels than required for its function under normal conditions [5,24]. Taken together, these observations make *Hsp83 *a good candidate for transposable element insertions in wild populations as well. The work we report below is the first to isolate such naturally occurring variants of *Hsp83 *and to characterize their effect on gene expression and fitness. Because *Hsp83 *has been associated with variation buffering, the question whether such mutants alter variation buffering is intriguing. We show that they indeed do.

Observations made with laboratory populations often do not apply to wild populations [42]. This holds especially when a population's evolutionary and genomic background matter, because wild populations have a history of inbreeding and selection that is different from laboratory populations [43]. For example, laboratory genetic mapping revealed a strong association between regulatory polymorphisms at the *hairy *locus and variation in sternopleural bristles. However, the same association does not exist in wild-caught flies [44]. A comparative study on 29 strains of *D. melanogaster *found that even central life history traits such as longevity can be highly strain-specific [45]. More generally, artificially selected phenotypes can only partly predict fitness components estimated in the field [46]. Thus, some observations derived from laboratory studies have dubious validity until confirmed also in the wild.

In this work, we first screened more than 4,500 flies from 42 world-wide *Drosophila *populations, and identified *P*-element insertions near the *Hsp83 *gene in three of these populations. We verified that these insertions indeed reduce *Hsp83 *gene expression. We then showed that flies with these mutations have lower competitive fitness, as well as lower fecundity and longevity. These effects occur in all three genetic backgrounds. We next used inbreeding experiments, which can increase homozygosity of deleterious alleles, to reveal cryptic deleterious mutations. These experiments showed that flies with reduced Hsp83 expression from all three populations can buffer deleterious variation much less well. Even mild environmental stress reduced the buffering so dramatically that populations went extinct. Experiments like these would have been impossible with strong laboratory mutations. The mild mutations we use allowed us to determine that wild-derived flies with even modest expression changes in the gene encoding Hsp90 show altered fitness and variation buffering that can be very strong on short evolutionary time scales.

## Results

### *Hsp83 *regulatory mutations exist in three out of 42 natural populations

**Figure 1:**
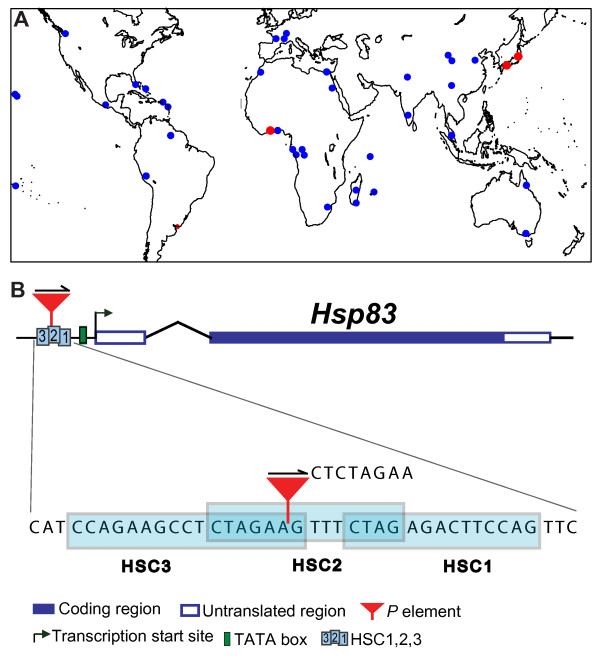
***D. melanogaster *sample locations, schematic gene structure of *Hsp83*, and the *P *element insertion site**. (A) Distribution of the natural fly populations collected around the world. Dots indicate the collection sites. The three red dots indicate the samples where a *P *element insertion into *Hsp83 *was found. The finnish population (F15 in additional file 1) is not shown. (B) Intron-exon organization of *Hsp83 *and the insertion of a *P *element into the proximal promoter of *Hsp83*. Below the *Hsp83 *gene structure an enlargement of the organization of *cis*-regulatory elements HSC1, HSC2 and HSC3 in the *Hsp83 *promoter region is shown. The 8-bp sequence (CTCTAGAA) flanking the *P *element is a direct repeat of the heat shock consensus elements created by the *P*-element insertion. The arrow above the *P *element shows the element's orientation. HSC, heat-shock consensus element (Xiao and Lis 1989). The structure is not drawn to scale.

In natural populations of *D. melanogaster*, the promoters of heat-shock genes are especially susceptible to the insertion of transposable elements. More than 96 percent of the transposable elements occurring in heat-shock promoters are *P*-element insertions. The promoter of *Hsp83 *is no exception [34,35]. For example, rates of transposable element insertions in single-copy heat shock promoters are estimated to be 14.7 times higher than the average rate of transposable element insertions in promoter regions of non-heat shock genes [34]. They are up to two times higher than the rate at which other insertion/deletions occur in non-coding regions of the fly genome [47]. Therefore, transposon insertion is a prominent candidate source of mutations for *Hsp83*, and makes such insertions an ideal object to study natural variation in *Hsp83*. To discover naturally occurring mutations in the *Hsp83 *promoter, we screened 42 strains derived from natural populations of *D. melanogaster *collected around the world (see the sample information in Figure 1A and in additional file 1). We aimed at discovering insertion/deletion mutations through electrophoretically detected length polymorphisms in PCR products amplified from the *Hsp83 *gene and its flanking regions. The PCR method we employed for *Hsp83 *gene-specific small fragment amplification (see Materials and Methods) is proven to be highly sensitive in detecting any insertion/deletion mutation. After having screened about 4500 flies from all 42 populations, we did not find any insertion polymorphism in the *Hsp83 *coding region. However, we did find insertion polymorphisms in the promoter region. They occurred in three separate populations, one from Okayama, Japan, a second population from Tokyo, Japan, and a third population from the Ivory Coast, Africa (See additional file 1).

Sequencing of the mutated gene region showed that the mutations were caused by insertion of non-autonomous *P *elements, i.e., the elements contain an internal deletion, and therefore cannot encode the functional transposase needed to mobilize the elements in genome. The *P *element insertions are identical in the three populations with respect to their insertion position, orientation and length as well as their flanking sequence (Figure 1B). The insertion is 1250 bp long and occurred 82 bp upstream of the transcription starting site of *Hsp83*. The inserted *P*-element disrupts the characterized regulatory sequences of *Hsp83 *[40,48]. Specifically, it integrated into an array of three heat-shock consensus elements that regulate *Hsp83 *expression [40,48]. Its integration moved one of these elements (HSC3 in Figure 1B) further away from the transcription start site, while at the same time creating an 8-bp (CTCTAGAA) direct repeat of the same element (Figure 1B). For further analysis, we established isogenic lines homozygous for the insertion mutation with individuals from each of the three populations where they occurred (Okayama, Tokyo, and Ivory Coast). We named their genotypes *Hsp83*^*P*/*P*^. We also established isogenic lines homozygous for the wild-type *Hsp83 *gene from the three populations, and named them *Hsp83*^*+*/*+*^. We note that the two Japanese populations and one African population had been collected in different years and maintained in different laboratories or stock centers (see Materials and Methods), thus making cross-contamination unlikely. Although the genotypes at the insertion site are identical, and may be derived from the same insertion event, the lines have different genetic backgrounds that result from the different geographic origin of their source populations.

### *P*-element insertion mutations reduce *Hsp83 *gene expression at both normal and elevated temperatures

**Figure 2:**
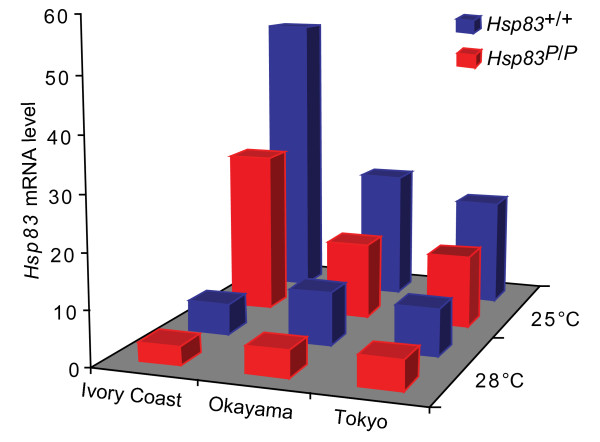
***Hsp83 *gene expression level in larvae of three different *D. melanogaster *populations**. Fly lines homozygous for the *Hsp83 *mutant (*Hsp83^P/P^*, red) and wild-type (*Hsp83*^+/+^, blue) strains were reared at two different temperatures (25°C and 28°C) for one generation before RNA was extracted. Flies with each genotype were isolated from three populations from Ivory Coast, Okayama, and Tokyo, respectively, as indicated. Note that *Hsp83 *expression in flies maintained at 28°C is downregulated compared with flies maintained at 25°C, which stands in contrast with *Hsp83 *expression in heat-shocked flies [40]. *Hsp83 *mRNA was purified from at least 20 early third-instar larvae, and quantified using real-time PCR. The expression of the housekeeping gene actin88F was used as an endogenous loading control. Expression levels of *Hsp83 *are measured relative to the expression of actin88F. Triplicate PCRs were performed for each mRNA sample.

Because the *P*-element integration disrupted this regulatory region, one would expect that this mutation affects *Hsp83 *gene expression. To validate this expectation, we performed real-time quantitative PCR to measure *Hsp83 mRNA *expression levels in third-instar larvae. We found that at normal temperatures (25°C) *Hsp83 *mRNA levels in mutants were reduced to 59.3 percent of the wild-type in the Okayama lines, to 68.0 percent of the wild-type in the Tokyo lines, and to 55.5 percent of the wild-type in the Ivory Coast lines (Figure 2). This reduction also extended to high temperatures. Specifically, flies cultured at 28°C for one generation decreased *Hsp83 *expression to 50.6 percent of the wild-type in the Okayama lines, to 62.7 percent of the wild-type in the Tokyo lines, and to 61.2 percent of the wild-type in the Ivory Coast lines. Cultivation at the higher temperature (28°C) generally decreased *Hsp83 *expression in both mutants and wild-type flies from the three populations (Figure 2).

These analyses were based on chromosomal alleles of *Hsp83*. It is possible that the observed changes in *Hsp83 *expression are not caused by these alleles, but by linked alleles at other loci that are in strong linkage disequilibrium with the *Hsp83 *alleles we study. To exclude this possibility, we isolated the wild-type and mutant *Hsp83 *promoter from the Okayama population and cloned them upstream of a luciferase gene. We then used the two resulting constructs to drive luciferase gene expression in transient transfection assays of two different *Drosophila *cell lines (S2R+ and Kc) [49] (See additional file 2A). In both cell lines, and both under normal temperatures and after heat shock at 37°C, the mutant promoter drove expression of the luciferase gene to a low level compared with the wild-type promoter. Specifically, the mutant promoter lowered luciferase gene expression between 11.9 percent (in non-heat shocked Kc cells) and 58.6 percent (in heat-shocked S2R+ cells; See additional file 2B). Taken together, these observations show that the *P*-element insertion mutants we study cause a reduced expression of the *Hsp83 *gene in three different genetic backgrounds. This reduction is caused by reduced activity of the mutant *Hsp83 *promoter, and not by indirect effects of alleles at loci linked to *Hsp83*.

### *Hsp83 *mutant alleles have low frequencies in natural populations

To estimate the frequencies of the two *Hsp83 *alleles (*Hsp83*^+ ^and *Hsp83^P^*) in the three source populations (Okayama, Tokyo, and Ivory Coast), we genotyped from 30 to over 300 flies from these populations (see Materials and methods). We found no mutant homozygotes in two of the populations, and only 0.3 percent of mutant homozygotes (i.e., one out of 312 individuals) in flies from the Okayama population. Among the flies we examined 3.2 percent, 5.5 percent and 15.4 percent were heterozygous for the *P*-element insertion in the Okayama, Tokyo and Ivory Coast populations, respectively. Expressed in terms of mutant allele frequencies in the sample of flies we examined, this means that the mutant *Hsp83 *allele had a low frequency of 1.5 percent in Okayama flies, 3.8 percent in Tokyo flies, and 7.7 percent in Ivory Coast flies. Only the genotype frequencies in the Okayama population significantly departed from Hardy-Weinberg equilibrium (χ^2 ^test, *p *= 0.037; See additional file 3).

We also attempted to identify nucleotide polymorphisms, but found only one polymorphism in a total of 3920 bp sequenced nucleotides, both upstream and downstream of the insertion site, in 23 flies of the Okayama population. These observations are consistent with previous work indicating that *Hsp83 *coding and regulatory regions show very little sequence polymorphisms [50].

The *Hsp83 *gene is located close to the breakpoint of the cosmopolitan inversion *Inv(3L)P *[51]. We wished to determine if any phenotypic effects of the Hsp83 mutation may have been indirect, caused by the inversion and its association (linkage disequilibrium) with the Hsp83 mutation. To this end, we scored the inversion *Inv(3L)P *polymorphisms in the two natural populations Okayama and Ivory Coast. We found no evidence of linkage disequilibrium with the Hsp83 alleles (Fisher's exact test, *p *> 0.05), and only rare inversion arrangements in flies with either Hsp83 genotype (See additional file 4). Thus, the phenotypic effects of Hsp83 mutations cannot be attributed to effects of the cosmopolitan inversion *Inv(3L)P*.

### *Hsp83 *transposon insertion mutants show lower fitness in competition assays

**Figure 3:**
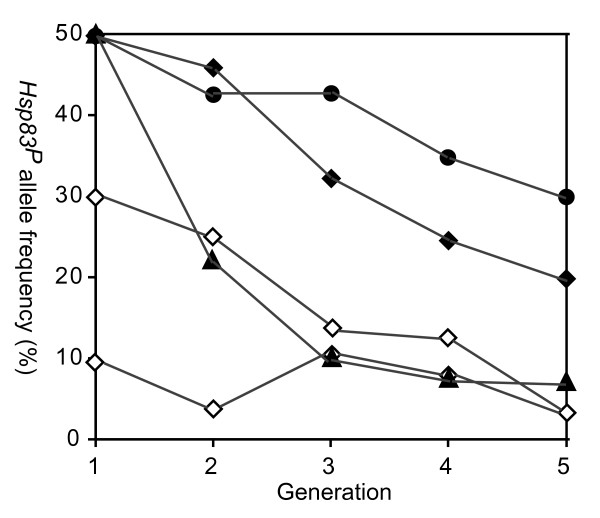
**Mutant flies have lower relative fitness in a co-culture competition assay**. We determined the relative fitness of mutant and wild-type individuals through a co-culture competition test lasting five generations at 25°C. For this test, we had obtained flies isogenic and homozygous for *Hsp83^P/P ^*and *Hsp83*^+/+ ^from the Okayama, Tokyo, and Ivory Coast populations, as described in the main text. We seeded three co-cultures with an equal number of flies of the mutant and wild-type genotype in three parallel co-culture competition experiments started with individuals from the Okayama (filled diamonds), Tokyo (filled cycles), and Ivory Coast population (filled triangles). We seeded two further co-culture competition experiments with lower percentages of mutant flies (30 and 10 percent, open diamonds) from the Okayama population. Each seeding population comprised 100 flies. We genotyped fifty flies in each generation. Note the consistent decrease in allele frequencies in most lines as time progresses.

The low population frequency of heterozygotes for the *Hsp83 *mutant allele, and in particular the near absence of mutant homozygotes in flies derived from wild populations, suggest that carriers of the mutant allele have lower fitness. To validate this observation we let wild-type and homozygous mutant flies compete with each other in a co-culture competition assay at 25°C. We performed two variants of this assay. In the first, we seeded populations with 50 virgin wild-type flies, and another 50 homozygous virgin mutant flies. Thus, in this assay, the initial frequency of the mutant allele was 50 percent. We propagated individuals from these populations for five generations (including the parental generation). In every single assay population, the mutant (*Hsp83^P^*) allele frequency decreased over the course of the experiment (Figure 3). Specifically, the *Hsp83^P ^*allele arrived at frequencies of 20, 30 and 7.5 percent after 5 generations in flies from the Okayama, Tokyo and the Ivory Coast populations, respectively. The three populations showed significant differences in the mutant allele frequency in co-culture (ANOVA: *F*_2,12 _= 41.242, *p *< 0.001), indicating that genetic background also has an influence on competition outcome.

The second variant of the assay that we performed involved only individuals from the Okayama population. It started with different proportions of mutant individuals. Specifically, it involved two starting populations, each consisting of 100 flies, where 30 and 10 flies, respectively, were initially homozygous for the *Hsp83^P ^*allele. In the first population *Hsp83^P ^*allele frequencies decreased by 89.3 percent. In the second population they fluctuated around the initial 10 percent. Both populations had a low *Hsp83^P ^*allele frequency of 3.2 percent at the end of this experiment.

In sum, our observations suggest that in populations where mutant flies constitute a substantial fraction of the population, they are competitively inferior to wild-type flies, and their population frequency declines over several generations. The number of individuals we used, as well as the consistent genotype frequency changes we observe in our replicate competition assays make genetic drift an unlikely sole cause of the changes we see.

### Mutant flies are less fecund and live less long, but are no less thermotolerant than wild-type flies

We next asked which component of fitness may be responsible for the reduced fitness of *Hsp83 *mutants. We investigated three fitness components. The first is fecundity. The second is longevity. The third is resistance to environmental high-temperature stress, which we studied because Hsp90 is known to be involved in thermotolerance.

**Figure 4:**
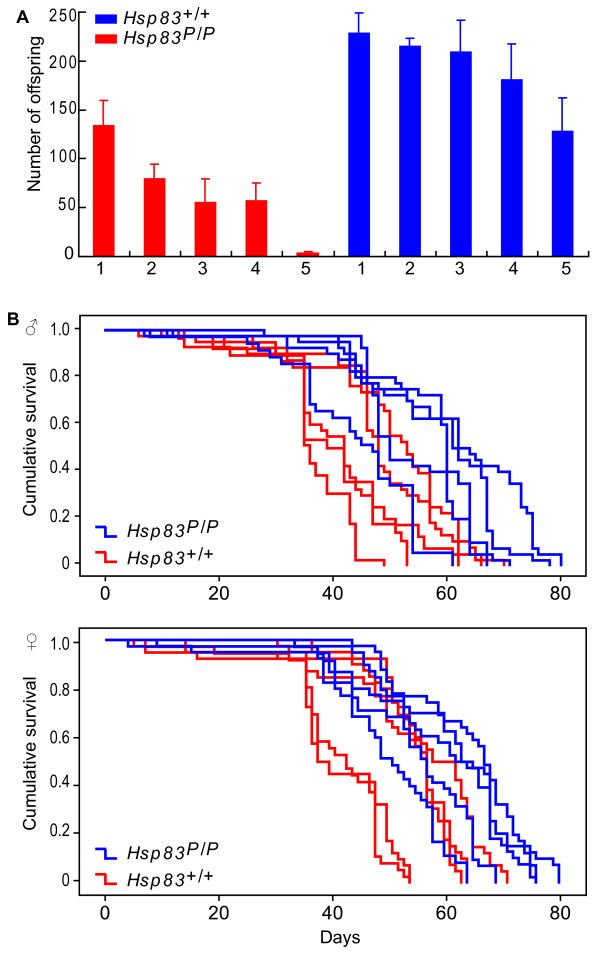
**Wild-type flies are more fecund and live longer than mutant flies**. (A) Female fecundity (eclosing adults per reproductive female) in *Hsp83 *mutant (*Hsp83*^*P*/*P*^) and wild-type (*Hsp83*^+/+^) isofemale lines. Each genotype is represented by five isofemale lines labeled one through five. Data for each isofemale line are derived from three replicate matings of five males and five females. Error bars above each column indicate one standard error of the mean derived from the three replicate matings. (B) Cumulative survival rate of male and females in *Hsp83 *mutant (*Hsp83*^*P*/*P*^) and wild-type (*Hsp83*^+/+^) isofemale lines. The horizontal axis shows the number of days after eclosion, the vertical axis the fraction of surviving individuals after a given number of days (i.e., the cumulative incidence of survival). Red and blue data were obtained from five isofemale lines of *Hsp83*^*P*/*P *^mutants and *Hsp83*^+/+ ^wild-type genotypes, respectively.

We first estimated female fecundity by counting all eclosing adults produced by females in isofemale lines obtained from the Okayama population (see Materials and methods). On average, wild-type females had significantly higher fecundity than mutant females (ANOVA: *F*_1,28 _= 20.841, *p *= 0.002). They produced three times more offspring than mutants, as shown in Figure 4A. The figure also indicates that different isofemale lines show significant differences in fecundity. A statistical test confirms this, and also demonstrates the non-negligible effect of genetic background on fecundity (Nested ANONVA: *F*_8,20 _= 3.166, *p *= 0.017, Figure 4A).

We next determined the longevity of mutant and wild-type genotypes. To this end, we cultured *Hsp83*^*P*/*P *^and *Hsp83*^+/+ ^lines in fresh medium at 25°C for the whole life of individuals in these lines (see Materials and methods), and counted the fraction of individuals that survived a given number of days after eclosion (Figure 4B). The mutant females had an average lifespan of 48.5 days, 14.9 percent shorter than the average life span of wild-type females at 56.9 days, a difference that was highly significant (Kruskal-Wallis Test: *F*_1,387 _= 5.194, *p *< 0.001). The mutant males showed a stronger reduction in average lifespan, with 43.2 days compared to 54.6 days in the wild-type. This difference of 20.9 percent is significant (Nested ANOVA: *F*_1, 370 _= 8.593, *p *= 0.019). Levene's test of error variances shows a significant difference in the variance of life span between wild-type and mutant lines in females (*F*_9,377 _= 4.086, *p *< 0.001), but not in males (*F*_9,370 _= 1.839, *p *= 0.060). This indicates that the *Hsp83 *mutation influences not only life span, but also its variability, at least for females. In sum, flies, especially males, are ageing faster when *Hsp83 *is mutated.

Lastly, we measured the basal thermal tolerance and inducible thermal tolerance of both the mutant (*Hsp83*^*P*/*P*^) and wild-type (*Hsp83*^+/+^) isofemale lines. We did not find any significant difference in basal or induced thermotolerance between the genotypes for either males or females. (*p *> 0.05; See additional file 5).

### Inbreeding shows that wild-type *Hsp83 *alleles buffer phenotypic expression of cryptic genetic variation better than mutant alleles

Inbreeding causes increased homozygosity at multiple genomic loci, and will thus expose phenotypic effects of recessive alleles to natural selection [52]. Such effects are usually deleterious. They are not apparent in outbreeding populations, where recessive alleles mostly exist in heterozygotes. In other words, outbreeding populations contain cryptic genetic variation-variation without phenotypic effects-at loci harboring recessive alleles. In inbreeding populations, this variation becomes phenotypically visible through its deleterious effects. One of the best-studied aspects of Hsp90 function in laboratory populations is the ability of Hsp90 to buffer the phenotypic effects of genetic variation [7,8,16,22]. With these observations in mind, we asked whether the natural Hsp90 mutants we study here are impaired in their ability to buffer cryptic variation that can be revealed by inbreeding. Such an observation would provide evidence that Hsp90's buffering ability is not just restricted to laboratory populations.

**Figure 5:**
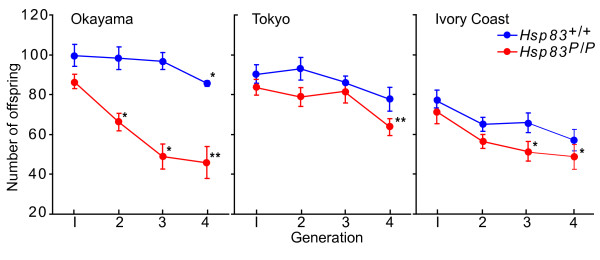
**Wild-type *Hsp83 *alleles can buffer cryptic deleterious variation caused by inbreeding**. Each panel shows changes in female fecundity (vertical axes) over 3-4 generations (horizontal axes) of inbreeding in fly populations containing either *Hsp83 *mutant or wild-type alleles. Seven lines homogenous for *Hsp83 *wild-type (*Hsp83*^+/+^) and for *Hsp83 *mutant (*Hsp83*^*P*/*P*^), respectively, were isogenized from each of three populations Okayama, Tokyo, and the Ivory Coast. In each generation, four virgin females and four males of an isofemale line were crossed to reproduce the next generation. All offspring produced within 30 days were counted as a measurement of female fecundity. Error bars indicate one standard error of the mean for the 7 isofemale lines. Independent-sample t-tests were performed to calculate the significance of fecundity difference between generation one, on the one hand, and generations 2-4, on the other hand. Asterisks indicate significant differences in fecundity to generation one: at *p *< 0.05(*) and *p *< 0.01(**).

To answer this question, we established 42 parallel inbred fly populations. Of these populations, 21 were homogenous for *Hsp83 *wild-type (*Hsp83*^+/+^), and 21 for *Hsp83 *mutant (*Hsp83*^*P*/*P*^) genotypes, respectively. More precisely, for each of the Okayama, Tokyo, Ivory Coast source populations, we established seven inbred populations with each of the two genotypes and propagated them at 25°C (see Materials and methods; Additional file 6). In each generation, we sampled 4 males and 4 females from each inbred population to create subsequent generations, and continued this process for 4 generations. As a measure of inbreeding depression-the deleterious effects of inbreeding on fitness-we estimated female fecundity by mating 4 virgin females and 4 males from each generation, and counted all offspring they produced within 30 days (See additional file 6). We now discuss results from each of the three lines in turn. First, in the inbred line from the Okayama population, wild-type females did not become significantly less fecund until generation 4 (*t *test: *p *= 0.051; Figure 5). In contrast, in the mutant line even one generation of inbreeding already caused a significant decrease in fecundity (*p *= 0.023), and by generation four fecundity had been reduced by 46.8 percent in the mutant. Second, in the inbred line from the Tokyo population (Figure 5), females did not become significantly less fecund in the wild-type after four generations (*p *> 0.05), indicating that the wild-type is well-buffered against short-term inbreeding. In contrast, females in the mutant population had become significantly less fecund after four generations (t test: *p *= 0.010; 23.2 percent reduction relative to the first generation). The Ivory Coast lines, finally, showed much lower initial fecundity than the other two inbred lines. Wild-type females showed no significant decrease in fecundity after four generations of inbreeding (*p *= 0.082), whereas mutants became significantly less fecund (*p *= 0.026, 30.7 percent reduction). In the four generations of inbreeding, the three populations showed significantly different fecundity within both the wild-type (*F*_2,80 _= 31.561, *p *< 0.001) and mutant genotypes (*F*_2,73 _= 12.880, *p *< 0.001). This indicates that effects of genetic background are important for our analysis and cannot be neglected. In sum, our analyses of inbred lines show that individuals with wild-type *Hsp83 *alleles buffer cryptic deleterious variation more effectively than mutant individuals. *Hsp83 *thus helps buffer cryptic deleterious variation in flies derived from wild populations.

### *Hsp83 *is even more important for cryptic variation buffering under mild heat stress

**Figure 6:**
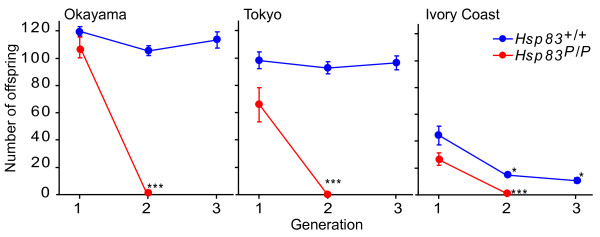
**Mild heat stress impairs the buffering ability of mutant *Hsp83 *enough to cause population extinction**. The horizontal axis shows time in generations after inbreeding lines had been established. The vertical axis shows the average number of offspring a female produces, i.e., female fecundity. Seven lines homozygous for wild-type *Hsp83 *(*Hsp83*^+/+^, blue) and for mutant *Hsp83 *(*Hsp83*^*P*/*P*^, red), respectively, were isogenized from each of the three geographic populations we studied here. From each isofemale line, four 3-day-old virgin females and four males were placed in a vial and allowed to mate to create the first generation. Subsequent generations were also produced from matings between four 3-day-old virgin females and four males. All offspring produced within 30 days were counted to estimate the fecundity per female. Error bars indicate one standard error of the mean among the 7 isofemale lines. Independent-sample t-tests were performed to ask whether fecundity differed between generation 1, on the one hand, and generation 2-3, on the other hand. Asterisks indicate significant differences at *p *< 0.05(*) and *p *< 0.001(***) obtained in these tests.

The preceding experiments showed that mutant *Hsp83 *alleles buffer cryptic deleterious variation to a lesser extent than wild-type alleles. We next asked whether high temperatures further exacerbate this difference. The rationale for this question is that Hsp90 is involved in the heat stress response. When exposed to heat stress, free Hsp90 will be diverted from its interactions with signaling proteins to help renature excess misfolded and aggregated proteins [1,24]. As a result, less Hsp90 protein may be available for signaling and buffering. To answer this question, we performed mild inbreeding at 28.0°C with the same methods for crossing of flies within inbred lines as used in the preceding experiments. The starting isofemale lines were also the same, except that we began to rear flies at 28.0°C when they eclosed (See Materials and methods; See additional file 6). In these experiments, wild-type flies from the Okayama and Tokyo populations retained high fecundity over three generations of inbreeding, while wild-type flies from the Ivory Coast populations significantly (*p *< 0.05) declined in fecundity after the first generation. All three populations persisted over three generations of inbreeding. In stark contrast to this persistence, lines with mutant *Hsp83 *alleles from the three populations went extinct after the first generation, because their fecundity had been reduced to almost zero (Fecundity loss significant at *p *< 0.001; Figure 6). This observation underscores that Hsp90 is important to buffer deleterious variation not only at normal temperatures, but even more so under thermal stress.

Outbreeding of a previously inbred population reduces homozygosity of deleterious recessive alleles. If our assertion that wild-type *Hsp83 *buffers deleterious variation is correct, then outbreeding should restore the fecundity lost during inbreeding, and especially in mutant lines where this loss is most severe (Figure 5 and 6). To ask whether this is the case, we used flies from the Ivory Coast population, where the fecundity loss had been especially severe at 28°C (Figure 6). We crossed flies derived from the different inbred isofemale lines with each other and examined their fecundity. In this way, we established populations that were outbred and contained either only *Hsp83 *mutant alleles, only wild-type alleles (See additional file 7A, "between-line" crosses), or a 1:1 mix of mutant and wild-type alleles (See additional file 7A, "between-genotype" crosses). The outbred populations show significant increases in fertility relative to their inbreeding controls (*p *< 0.05). Specifically, outbreeding of populations that contained only mutant alleles (*Hsp83*^*P*/*P *^× *Hsp83*^*P*/*P *^in "between-line" crosses) increased fecundity from zero (after inbreeding at 28.0°C) to 12 individuals per female (Figure 6 and S4B). This change in fecundity was highly significant (*p *< 0.001), indicating that outbreeding rescued the fecundity loss during inbreeding at mild heat stress. Wild-type alleles of *Hsp83 *helped increase fecundity even further. Specifically, the fecundity of outbred populations with both allele types (*Hsp83*^+/+ ^× *Hsp83*^*P*/*P*^; "between-genotype" cross in additional file 7B) was 167 percent higher than for populations with only the mutant alleles (*Hsp83*^*P*/*P *^× *Hsp83*^*P*/*P*^; *p *= 0.018). Outbreeding in populations with only wild-type alleles (*Hsp83*^+/+ ^× *Hsp83*^+/+^) increased fecundity by an additional 70 percent, to a level that was significantly higher than in the mixed cross (*Hsp83*^+/+ ^× *Hsp83*^*P*/*P*^; *p *= 0.013) (See additional file 7B). In sum, wild-type Hsp83 alleles are much more effective in restoring the fecundity that is lost after inbreeding.

## Discussion

We discovered naturally occurring mutants of the *Hsp83 *gene with *P *element insertions in the gene's proximal promoter region. These mutants occur in three out of 42 populations that we examined, and at modest allele frequencies, not exceeding 7.7 percent. The mutations down-regulate *Hsp83 *gene expression by 32 percent to 40 percent, depending on the population. They reduce competitive fitness, female fecundity, and longevity. We also found that *Hsp83 *(and possibly linked loci) strongly influences the expression of cryptic deleterious genetic variation in inbred populations. That is, flies that carry the mutant *Hsp83 *allele are much more poorly buffered against such variation than flies with the wild-type allele. We found that even mild thermal stress can completely break down the impaired buffering associated with mutant *Hsp83*. Specifically, inbred fly populations with mutant *Hsp83 *alleles go extinct at modestly elevated temperatures of 28°C.

To our knowledge, this work is the first to show that naturally occurring *regulatory *mutations of the *Hsp83 *gene-a key modulator of the stress response and cellular signaling-can affect reproductive success, reduce longevity, and reduce variation buffering in fruit flies derived from wild populations. A previous study on naturally occurring *Hsp83 *variants had identified a nonsynonymous deletion mutation in the *Hsp83 *coding region, and focused on the effects of this mutation on morphological traits, not on fitness components [27]. In addition, this study perturbed variation buffering only through thermal stress exposure, not through inbreeding, which is especially useful to reveal expression of recessive cryptic genetic variation. Our limited sequence polymorphism analysis found only one synonymous mutation in the *Hsp83 *coding region in the flies we studied, thus making it unlikely that the same deletion mutation stands behind our observations.

Our naturally occurring variants of *Hsp83 *have fairly mild effects compared to some of the drastic perturbations that earlier laboratory experiments used [7,11,16,22,26,32,33]. For example, the reduced expression of *Hsp83 *in our mutants did not significantly affect thermotolerance in outbred flies (See additional file 5). Working with these mutants has several benefits. First, it avoids pharmacological manipulation or structural modification of Hsp90, both of which may have unknown side effects, for example, by changing the protein's molecular interaction partners [1,25]. Second, it avoids the use of mutations with drastic expression effects, such as engineered homozygous loss-of-function mutants [31,32]. Such mutants can only be maintained as heterozygotes in the laboratory [16,33]. A continuous inbreeding experiment like the one we performed to reveal deleterious cryptic variation would be difficult with such drastic mutations, partly because homozygote offspring would be lethal. In contrast, the mild variants we found enabled us to ask whether more *Hsp83 *means better buffering [53]. Finally, they allowed us to study flies derived from natural populations, and to examine whether laboratory findings on Hsp90 apply to such populations.

A potential disadvantage of working with natural populations and the mutants that they contain is that each mutant occurs in a different genetic background, and that this background can influence observations. For example, we found that the genetic background of each geographic population influenced the competitive ability and fecundity of the flies we studied. However, our key observations, for example that *Hsp83 *can buffer deleterious variation in inbreeding lines, and that *Hsp83 *mutants show reduced fitness, were consistent across all three genetic backgrounds.

*P *element insertions are widespread in the *Drosophila *genome, and they are especially abundant in heat-shock genes [34,35]. In Japanese natural populations, for example, at least 6 distinct *P *element insertions exist in the heat shock gene *Hsp26*, and at least 5 distinct insertions exist in the heat-shock gene *Hsp27 *at high population frequency [35]. These observations stand in stark contrast to the low frequency of insertions into the *Hsp83 *gene. They suggest that the low frequency of *P*-element insertions we observe in *Hsp83 *are not a consequence of a recent insertion, but that it results from natural selection against mutant *Hsp83*. Our observation that these insertions are possibly associated with reduced competitive fitness, reduced fecundity, and shorter life span are fully consistent with this suggestion. The question then arises why we observe *any *individuals that carry *P*-element insertions in our study populations. One possible answer is that the insertion may be neutral or even beneficial when heterozygous or when at low population frequencies. Our observation that mutant allele frequencies did not decline in a competition assays seeded with only 10 percent of *Hsp83^P ^*alleles are consistent with this possibility. Moreover, the fitness effect of *Hsp83^P ^*may depend on the environment to which a population is adapted, as has been observed for *P *element insertions in other heat shock genes [35,54]. In addition, some *P*-element insertions can help maintain tradeoffs between stress resistance and developmental homeostasis of flies living in a changing environment [34,35,37]. Unfortunately, our data does not allow us to answer which of these possibilities is responsible for the maintenance of *P *element insertions at modest frequencies in our study populations.

Our observation that mutations in *Hsp83 *affect fecundity is consistent with other reports that *Hsp83 *is involved in reproductive functions. *Hsp83 *plays a critical role in the process of both oogenesis [55] and spermatogenesis [56]. For example, *Hsp83 *RNA is a component of the posterior polar plasm [57], and Hsp90 protein is required for localization of maternal mRNA to the posterior pole, which is essential for development of germ cells in the *Drosophila *embryo [58]. Thus, *Hsp83 *is involved in the molecular pathways responsible for oogenesis and spermatogenesis in *D. melanogaster*.

Inbreeding increases the fraction of homozygous loci in a genome. Because many alleles are recessive and deleterious in the homozygous state [59], inbreeding will cause previously cryptic (heterozygous) deleterious variation to be expressed [52]. Such deleterious variation manifests itself as a reduction in one or more fitness components, such as fecundity. In our inbreeding experiments, we found that wild-type *Hsp83 *flies were better buffered against the deleterious effects of inbreeding than mutant flies. Specifically, wild-type *Hsp83 *flies from all three geographic populations showed no decline in fecundity after three generations of inbreeding, and only one population showed a small decline (by 14 percent) after four generations. In stark contrast, mutant lines from all three geographic populations showed a significant decline in fecundity of up to 47 percent after between two and four generations. Genetic polymorphisms are widespread in many populations [47,59], but the incidence of cryptic variation is usually unknown for wild populations [14,15]. Inbreeding can reveal such variation. Our experiments demonstrate that cryptic variation must be abundant in the populations we studied, because inbreeding reduced fecundity substantially. Moreover, the experiments show that expression of *Hsp83 *at wild type levels can buffer the damage caused by inbreeding.

The observation that a chaperone can buffer deleterious variation is not unprecedented. Over-expression of GroEL, a molecular chaperone of *Escherichia coli*, can help overcome the accumulation of deleterious mutations that occur in *E. coli *strains with high mutation rate [20]. In other words, a chaperone can buffer these organisms against deleterious mutations [17,20]. Although the sources of deleterious variation-inbreeding and mutation accumulation-and the chaperones-Hsp90 and GroEL-differ in these two organisms, chaperones have the same qualitative effect in both cases. However, the *E. coli *strain in which these previous experiments were conducted is a laboratory strain. Observations made with this strain need not necessarily transfer to populations in the wild.

Three main observations lead us to think that the buffering capacity of Hsp90 is relevant for wild fly populations. First, the flies in which we identified the effects of the *Hsp83 *mutation all stem from wild populations. Second, although the buffering capacity of Hsp90 varies with genetic background, significant buffering did occur in all three populations. Third, the regulatory mutation in *Hsp83 *(and possibly linked loci) is mild in its effect on expression, but strong in its effects on fitness, i.e., on fecundity, longevity, and most importantly, buffering capacity. Mutations that affect these aspects of fitness are likely to be selected against in nature [59]. More specifically, because *Drosophila *has very large effective population size in the wild [43], fitness differences much smaller than we detected would be visible to natural selection [14,59], and would lead to the eventual elimination of mutant alleles with low fitness.

## Conclusions

In sum, our study shows that natural variants in the *Hsp83 *gene can affect the fitness of flies derived from natural populations, regardless of their genetic background and geographic origin. It also shows that *Hsp83 *is involved in the buffering of deleterious variation in these flies, and that this role becomes even more important under heat stress. And it demonstrates that Hsp90 plays an important role in the life of flies in the wild.

## Methods

### *D. melanogaster *strains

The 42 populations of *D. melanogaster *we examined were established from wild-caught flies sampled in multiple locations around the world (see the sample information in Figure 1A and additional file 1). Population F1 is represented by 24 isofemale lines from flies collected in Okayama, Japan, in 2004 (reference numbers OKY1-24 in EHIME-Fly, the *Drosophila *stock center of Ehime university; http://kyotofly.kit.jp/cgi-bin/ehime/index.cgi). Population F2 is represented by 24 isofemale lines that were collected in Tokyo, Japan in 2003 (reference numbers OGSC1-24 in EHIME-Fly). Dr. Wenxia Zhang at Peking University, China provided individuals from populations F3-F6, which are derived from natural populations in China. Population F7 is represented by five isofemale lines from Cairns, Australia, and population F8 by 13 isofemale lines from Melbourne, Australia. Flies from the populations F7 and F8 were obtained from Dr. Ary A. Hoffmann at the University of Melbourne. Individuals from populations F10-11, F19 and F24-25 were obtained from the Tucson *Drosophila *Species Stock Center (reference number: 14021-0231.06, 14021-0231.04, 14021-0231.00, 14021-0231.20, 14021-0231.01; https://stockcenter.ucsd.edu). Individuals from the other 29 natural populations were obtained from Dr. Jean David in CNRS, France, and have been the subject of previous investigations [34,60,61]. All live flies for this study were reared on a medium of cornmeal, molasses, and agar at a temperature of 25°C, a humidity of 60 percent, and light period of 12D:12L in an environment-controllable incubator (*Adaptis *A1000 manufactured by CONVIRON, Canada; http://www.conviron.com).

### Screening *D. melanogaster *populations for mutations in *Hsp83*

To identify insertion/deletion (indel) mutations in *Hsp83 *and its flanking regions, we first designed three primer pairs to amplify three overlapping regions covering *Hsp83 *and the 5' part of the gene CG14965 immediately upstream of *Hsp83*, as well as the entire gene CG14966 immediately downstream of *Hsp83 *(see additional file 8 and sequences in additional file 9 of the 3 primer pairs (i) CG14965-S1F and S1R, (ii) S2F and S2R, (iii) S3F and CG14966-S3R). With these primers we could detect indels through the polymorphisms they yield in the size of PCR amplification products. Because indel mutations preferentially occur in *Hsp *promoter regions [34], we also designed a primer pair to specifically amplify the intergenic region upstream of *Hsp83*. This pair consists of primer CG14965-S4F, which is complementary to DNA in the upstream gene CG14965, and S4R, which is complementary to part of the 5' region of *Hsp83 *(See additional file 8 and additional file 9). We sequenced amplicons obtained from this primer pair that were larger than expected from the wild-type sequence (1156 bp), in order to confirm the identity, structure, position, and orientation of insertions into the promoter. We also applied a PCR that can specifically detect *P *elements in the *Hsp83 *promoter region to flies from lines where previous PCR analysis had revealed insertions. For this PCR, we used the forward primer PIA8, which is specific to the flanking inverted repeats of *P *elements, and the primer S4R (See additional file 8 and additional file 9). We estimated allelic frequencies of *P *element insertions in *Hsp83 *via the above two amplification reactions, i.e, one for indel detection in the intergenic region, another for the *P *element-specific amplification. Overall, we screened approximately 100 *D. melanogaster *individuals from each of the 42 populations for *Hsp83 *indels, for a total of more than 4,500 screened flies. We purified genomic DNA from individual adult flies with the Puregene DNA purification system (Gentra Systems) according to the manufacturer's instructions.

### Polymorphisms in *Hsp83 *nucleotides and the inversion *Inv(3L)P*

We examined single nucleotide polymorphisms in a 940 base pair (bp) long stretch upstream of the insertion site in 14 mutant flies from the Okayama population, and found only one point mutation in a single sequence. Primer pair PIA8 and CG14965-S1F was used for the amplification (See additional file 8 and additional file 9). In nine flies of the same population, we also examined nucleotide polymorphisms in a 2980 bp stretch downstream of the insertion site amplified with primer pair PIA8 and CG14966-S3R (See additional file 8 and additional file 9). This region covers the whole *Hsp83 *coding region and most of the downstream gene *CG14966*. This analysis found no polymorphisms.

To determine if the *Hsp83 *mutation was in linkage disequilibrium with the inversion *Inv(3L)P*, we genotyped the *Hsp83 *mutant and wild-type lines for the presence of the inversion following an established method [27]. Briefly, in this method each DNA sample is genotyped by two primer pairs, one yielding a 1350 bp fragment for the standard arrangement, and another pair yielding a 1075 bp fragment for the inversion. To exclude the possibility that polymorphisms in priming sites yield artifactual results, we also used another genotyping method [62] in which two different primer pairs detect the standard and inverted arrangements. We genotyped 25 flies in Okayama lines and 26 flies in Ivory Coast lines using both methods.

### Construction of isogenized homozygous lines

Virgin females were collected from each of the three populations in which *P *element insertions existed in *Hsp83 *promoters. Individual females were paired with one male and allowed to mate in vials containing 7 ml of standard medium at 25°C. Adult offspring were removed after 5 days and genotyped as described above. Offspring of heterozygous parents were allowed to mate and reproduce further until pairs of lines homozygous for a *P *element insertion (*Hsp83*^*P*/*P*^) and for the wild-type *Hsp83 *promoter (*Hsp83*^+/+^) were established in the same generation. Individuals with the *Hsp83*^*P*/*P *^or *Hsp83*^+/+ ^genotypes were then mated inter se to obtain large populations of more than 200 individuals. Pair-wise mating among individuals of the two genotypes was undertaken during the population maintenance, so that the mutant and corresponding wild-type lines used in the next experiments had the same history of inbreeding, and did not differ in average levels of genetic variation. These populations of isofemale lines were maintained for two generations before the experiments described below, in bottles at a constant temperature of 25°C, humidity of 60 percent, and light period of 12D:12L in an incubator. Homozygous lines of the two genotypes used for the measurement of fecundity, longevity, and thermal tolerance were all isogenized from the Okayama population (See additional file 10).

### Quantitation of *Hsp83 *gene expression by real-time PCR

Total RNA was purified from about 25 early 3^rd ^instar larvae using an RNeasy Protect kit (Qiagen, Germany) according to the manufacturer's instructions. Equal amounts (1 ug) of RNA were used to synthesize first-strand cDNA with an Oligo(dT) primer using a reverse transcription kit (ABgene, Rochester, NY, USA). The housekeeping gene actin88F was used as an endogenous control to calibrate total mRNA levels. The mRNA level expressed by a specific gene was measured by real-time quantitative PCR on an Applied Biosystems 7500 Fast Real-Time PCR System with SYBR^® ^Green I dye chemistry (ABgene, USA). Measurements were carried out in three technical replicates for each mRNA sample. The relative quantification of gene expression of *Hsp83 *and actin88F was calculated by the comparative cycle threshold method (ΔΔCT Method) according to ABI's guide (Applied Biosystems, USA). Primers used for quantitative PCR are listed in additional file 9.

### Construction of luciferase reporter plasmids, transient transfection, and dual luciferase assay

DNA fragments containing *Hsp83 *wild-type and *P*-element insertion promoters were PCR-amplified from isofemale lines derived from the Okayama population (See additional file 1, population F1). These fragments were cloned into the promoter/enhancer-free pGL-3 basic vector (Promega), which contains a luciferase gene. To facilitate cloning, the PCR used primers (Kpn1F1, Bgl2R1; See additional file 9) designed to introduce restriction sites into the amplified fragments. The amplified region begins 714 bp upstream and ends 35 bp downstream of the transcription start site (See additional file 2A). The methods for construction of luciferase reporter plasmid were the same as in [35].

Two types of *Drosophila *cells, S2R+ and Kc cells [49], were transfected with pGL3-basic vector DNA with insert, or without insert for control experiments in 96-well plates. Both a firefly luciferase reporter gene construct (200 ng) and a pRL-SV40 *Renilla *luciferase construct (10 ng; for normalization) were co-transfected in each well. After transfection, cells were incubated at 25°C for 12 h, placed in a cell incubator at either 36.5°C (heat shock) or 25°C (control) for 5 min, transferred to a 25°C cell incubator for 30 min, and harvested by centrifugation. The luciferase activity was then measured using the Dual-Luciferase Reporter Assay System (Promega, USA) according to the instruction manual. Four replicate cell lines were prepared and assayed for each treatment. The intensity of the firefly luciferase signal is reported here as the ratio of averaged firefly to Renilla luciferase luminescence.

### Competition assay for relative fitness determination

Relative fitness of *Hsp83 *wild-type (*Hsp83*^*+*/*+*^) and *P*-element insertion lines (*Hsp83*^*P*/*P*^) was measured in a bottle with competing flies of both genotypes. To make sure that the relative competitive ability of the parental females in this experiment was due to genetic rather than environmentally induced effects, all parental flies were raised at the same sex ratio (1:1), population density (~150 flies per bottle), age, temperature (25°C) and humidity (60 percent). Two different types of competition assays were performed to measure relative fitness. The first used a population of competing flies with an initial 1:1 ratio of the two genotypes, i.e., with 50 *Hsp83^P/P ^*and 50 *Hsp83*^*+*/*+ *^virgin adults. For this assay, three parallel competition populations were established with flies derived from three different populations, one from Okayama (population F1 in additional file 1), one from Tokyo (population F2), and one from the Ivory Coast (population F3). The second assay used three populations of competing flies with different proportions of mutant and wild-type flies from the Okayama population. The three competition populations initially contained 10 percent, 30 percent, and 50 percent homozygous *P*-insertion genotypes. Specifically, the first population was established by mixing 10 *Hsp83^P/P ^*with 90 *Hsp83*^*+*/*+ *^adults, the second population by mixing 30 *Hsp83^P/P ^*with 70 *Hsp83*^*+*/*+ *^adults, and the third by mixing 50 *Hsp83^P/P ^*with 50 *Hsp83*^*+*/*+ *^adults.

From each of these populations flies were transferred every two days to a new bottle to lay eggs. One hundred virgin offspring flies randomly taken from over 600 flies grown in bottles from three consecutive time points, each between three and seven days old, were mated to found the next generation. All populations were cultured for 5 consecutive generations. Adult offspring in each generation were frozen for genotyping after they laid eggs. Thirty to fifty flies of males and females were genotyped in each generation. Since no difference in the frequency of alleles that carry *P*-element insertions was observed between males and females, the allele frequency was calculated for a mix of both sexes. The change in frequency of *P*-element carrying alleles was used to estimate the relative fitness of individuals with such insertions.

### Fecundity

Five isofemale lines homozygous for the insertion (*Hsp83^P/P^*) and five male lines homozygous for the wild-type (*Hsp83*^+/+^), all of them established from the Okayama population, were subjected to the following procedure. Five females were collected as virgins and placed, one day after eclosion, with five males in glass vials containing 7 ml of standard medium to which powdered yeast had been added. The ten flies were then transferred to a fresh vial every 48 hours until no eggs had been laid in two consecutive seeding vials. Adults eclosing from each vial were censused daily until eclosion ceased. All offspring from each mating were counted as a measurement of female fecundity. This procedure was carried out three times independently for each of the 10 lines. Fecundity measurements in inbreeding lines were performed as described below.

### Longevity

Five isofemale lines homozygous for *Hsp83*^+/+ ^and five homozygous for *Hsp83*^*P*/*P *^were used for the longevity measurement. Forty flies of the same sex and same age from each of the ten lines were reared in glass vials containing 7 ml of standard medium, and transferred to new medium every two days. During this time, dead flies were counted and removed. This procedure (transfer, counting and removal of dead flies) was continued until all flies had died.

### Thermal stress tolerance

To determine basal thermal tolerance, three-day-old adults were heat-shocked in a 40.3°C water bath for 30 minutes, and survival was checked after 24 hours' recovery at 25°C. To determine induced thermal tolerance, 3-day old adults were first pretreated at 29.0°C for 20 minutes, followed by a 40 minute treatment at 36.5°C to maximally induce *Hsp83 *expression [40], and a subsequent 30 minute heat-shock treatment at 40.3°C. For these treatments, samples of about 30 flies were placed in glass vials containing 7 ml of standard medium, and the vials were submerged in water baths at the temperatures described. Flies were tallied as survivors if, after 24 hours at 25°C, they responded to gentle brush prodding. The flies subjected to these treatments came from each of 13 to 20 Okayama isofemale lines, and included individuals of both sexes.

### Continuous inbreeding and outbreeding

From each of the three natural populations from Okayama, Tokyo, and Ivory Coast (See additional file 1), seven isogenic lines (Iso-1 to Iso-7) homozygous for *Hsp83 *wild-type (*Hsp83*^+/+^) and for *Hsp83 *mutants (*Hsp83*^*P*/*P*^), respectively, were established in the standard isogenization method described above. Four 3-day-old virgin females and four 3-day-old males of an isofemale line were mated in a vial to generate the first generation. The 8 flies in the vial were transferred to a new vial with fresh food every other day. The transfer continued for 30 days or until no hatched larvae were visible in the vial. Adults eclosing from each vial were censused every other day until eclosion ceased. To generate the second generation, four females and four males collected on the sixth eclosing day within an isofemale line were sexed, separately reared for 3 days, and then mated in a vial to lay eggs. Using this protocol, the inbreeding experiments proceeded for 4 generations at 25(± 0.2)°C, and for 3 generations at 28.0(± 0.2)°C. At the higher temperature, the mutant lines ceased to reproduce in the second generation (See the experimental design in additional file 6). All offspring from each quadri-mating were counted to estimate female fecundity.

For the outbreeding experiment, virgin adult offspring of the two genotypes (*Hsp83*^+/+ ^and *Hsp83*^*P*/*P*^) derived from the second generation of the above inbreeding experiment at 28°C were collected from all seven isofemale lines of the Ivory Coast population (Iso-1 to Iso-7). They were then outbred at 28°C according to the experimental design shown in additional file 7. Specifically, two different outbreeding regimes were applied. First, to investigate if relieving inbreeding can restore the fertility loss caused by *P*-element insertion into *Hsp83 *under stress, flies from two of the seven isofemale lines within each genotype were crossed. In total seven crosses were established from the seven inbred isofemale lines within each genotype. Second, in an attempt to rescue the *Hsp83 *down-regulation in mutant lines, flies of two lines from each of the two genotypes were crossed. Since Hsp90 is essential for both oogenesis and spermatogenesis [55,56,63,64], between-genotype crosses were only performed between mutant females and wild-type males, but not reciprocally. Overall, seven between-genotype crosses were established from flies of the seven inbred isofemale lines. In each cross, four 3-day-old females and four 3-day-old males were mated in a vial. The eight flies in the vial were transferred to a new vial with fresh food every other day for a total of 30 days. Adults eclosing from each vial were censused every other day until eclosion ceased. Then female fecundity was measured by examining the number of offspring from each cross as described in previous sections.

### Data analysis

For longevity, fecundity and thermal tolerance, the effects on the mean phenotype of genotype (*Hsp83^P/P ^*and *Hsp83*^*+*/*+*^) and line within each genotype were tested using a nested one-way analysis of variance with genotype as a fixed factor, and the line nested within genotype as a random variant. Separate ANOVAs were performed for males and females. A Kruskal-Wallis nonparametric test was performed instead when the variances are different between genotypes. Independent-sample t-tests were performed to examine whether fecundity differed significantly between the first generation on the one hand, and the second to fourth generation of inbreeding at 25°C and 28°C, on the other hand. Levene's test [65] was used to determine if a given number *k *of samples had equal variances.

## Authors' contributions

BC carried out the experiments. BC and AW conceived of and designed the study, performed the data analysis, and drafted the manuscript. Both authors read and approved the final manuscript.

## Supplementary Material

Additional file 1 **Table S1. The 42 natural populations of *D. melanogaster *screened for *Hsp83 *insertion/deletion mutations**. Lines F1, F2, F7, and F8 were reared as isofemale lines. The remaining lines were mass-reared. Click here for file

Additional file 2 **Figure S1. *P *element insertion mutation in the *Hsp83 *proximal promoter reduces *Hsp83 *gene expression**. (A) *Hsp83 *gene promoter-luciferase reporter constructs (see also Materials and methods). Promoters of the *Hsp83 *gene (both as wild-type and with *P*-element insertion) from the Okayama population were amplified, digested with Kpn1 and Bgl2, and ligated into the promoter region of the luciferase gene in the pGL3-basic vector as described in Materials and Methods. HSC, Heat-shock consensus element; TATA, TATA box. Constructs are not drawn to scale. (B) *Hsp83 *gene promoter activity, as measured by a luciferase assay (see also Materials and methods). *Drosophila *S2R+ cells and Kc cells were transiently transfected in separate experiments with luciferase constructs that contained the wild-type *Hsp83 *promoter, and the same promoter with an inserted *P *element. Transfectants were subject to control (i.e., non-heat-shock) or 37°C heat-shock treatments, as described in Materials and methods. The vertical axis shows the firefly luciferase signal, expressed as the ratio of firefly to Renilla luciferase luminescence measured from 4 replicate cell lines for each treatment. Click here for file

Additional file 3 **Table S2. Frequency of *Hsp83*^*P*/*P *^and *Hsp83*^*P*/+ ^alleles occurring in three populations**. Click here for file

Additional file 4 **Table S3. No association between frequency of the inversion *Inv(3L)P *and *Hsp83 *alleles**. Click here for file

Additional file 5 **Figure S2. Mutant flies are no less thermotolerant than wild-type flies**. We determined thermal stress tolerance via adult mortality of males (upper panel) and females (lower panel) under heat-shock stress in mutant and wild-type lines. We determined basal thermal tolerance (labeled as 'Basal' in the figure) by heat-shocking 3-day old adults in a water bath at 40.3°C for 30 minutes, and counted survivors after a 24 hour recovery period at 25°C. We measured induced thermal tolerance (labeled as 'Induced') by pre-treating flies at 29.0°C for 20 minutes, followed by 40 minutes at 36.5°C for *Hsp83 *expression induction, before heat-shocking flies as above at 40.3°C for 30 minutes. We subjected about 30 flies of each genotype (*Hsp83*^*P*/*P *^and *Hsp83*^+/+^) from each of 13 to 20 isofemale lines to this treatment. Error bars indicate one standard error of the mean. Click here for file

Additional file 6 **Figure S3. Experimental design for continuous inbreeding of flies at 25°C and 28°C**. Flies from 7 isofemale lines for each genotype (wildtype *Hsp83*^+/+ ^vs. mutant *Hsp83*^*P*/*P*^) were subject to the same inbreeding procedure in parallel. In each generation, four 3-day-old virgin females and four 3-day-old males of an isofemale line were mated in a vial to generate the next generation. The 8 flies in the vial were transferred to a new vial with fresh food every other day for a total of 30 days. Adults eclosing from each vial were censused every other day until eclosion ceased. At 25°C inbreeding continued for 4 generations. At 28°C inbreeding continued for 3 generations for wild-type flies, and for 2 generations of mutant flies, because the flies ceased to lay eggs in the second generation. Fly lines isogenized from each population were subject to the same inbreeding procedure. Click here for file

Additional file 7 **Figure S4. Outbreeding rescued the fertility loss caused by stress at 28°C in *Hsp83 *mutants**. (A) Experimental design for outbreeding of flies at 28°C. Flies from 7 isofemale lines (iso-1 to iso-7) for each genotype (wildtype *Hsp83*^+/+ ^vs. mutant *Hsp83*^*P*/*P*^) were collected from adults eclosed in the second generation of inbreeding at 28°C for the Ivory Coast population (see the inbreeding design in Materials and methods and additional file 6). Virgin females from one line and males from another line with the same genotype were mated to establish between-line crosses. In addition, virgin females from one mutant line and males from a wild-type line were mated to set up between-genotype crosses. In total, seven between-line crosses for each genotype and seven between-genotype crosses were established. In each cross, four 3-day-old females and four 3-day-old males were mated in a vial. The eight flies in the vial were transferred to a new vial with fresh food every other day for 30 days. Adults eclosing from each vial were censused every other day until eclosion ceased. × indicates crossing. (B) female fecundity from crosses between lines and crosses between genotypes. Independent-sample t-tests were performed to calculate significance of fecundity difference between two crosses. Asterisks (*) indicate significant differences at *p *< 0.05 in these tests. Click here for file

Additional file 8 **Figure S5. Position of primers located in the chromosome region containing the genes CG14965, *Hsp83*, and CG14966**. Filled areas correspond to genes (black bars) and to a *P *element (red triangle). The tapered ends of black bars indicate the direction in which a gene is transcribed. Primers are described in Materials and methods. The arrow near each primer indicates the primer's 5' > 3' direction. Primer sequences are shown in additional file 1. The sequence is drawn to scale. Click here for file

Additional file 9 **Table S4. Sequences of the primers used in Materials and Methods**. Click here for file

Additional file 10 **Table S5. Fly populations and isofemale lines used for trait measurements**. Click here for file

## References

[B1] Young JC Moarefi I Hartl FU Hsp90: a specialized but essential protein-folding tool J Cell Biol 2001 154 267 274 10.1083/jcb.200104079 11470816 PMC2150759

[B2] Hartl FU Molecular chaperones in cellular protein folding Nature 1996 381 571 580 10.1038/381571a0 8637592

[B3] Walter S Buchner J Molecular chaperones-cellular machines for protein folding Angew Chem Int Ed 2002 41 1098 1113 10.1002/1521-3773(20020402)41:7<1098::AID-ANIE1098>3.0.CO;2-9 12491239

[B4] Peterson FC Baden EM Owen BA Volkman BF Ramirez-Alvarado M A single mutation promotes amyloidogenicity through a highly promiscuous dimer interface Structure 2010 18 563 570 10.1016/j.str.2010.02.012 20462490 PMC2872106

[B5] McClellan AJ Xia Y Deutschbauer AM Davis RW Gerstein M Frydman J Diverse cellular functions of the Hsp90 molecular chaperoneuncovered using systems approaches Cell 2007 131 121 135 10.1016/j.cell.2007.07.036 17923092

[B6] Wagner A Robustness and evolvability in living systems 2005 Princeton: Princeton University Press

[B7] Queitsch C Sangster TA Lindquist S Hsp90 as a capacitor of phenotypic variation Nature 2002 417 618 624 10.1038/nature749 12050657

[B8] Jarosz DF Lindquist S Hsp90 and environmental stress transform the adaptive value of natural genetic variation Science 2010 330 6012 1820 1824 10.1126/science.1195487 21205668 PMC3260023

[B9] Hermisson J Wagner GP The Population Genetic Theory of Hidden Variation and Genetic Robustness Genetics 2004 168 2271 2284 10.1534/genetics.104.029173 15611191 PMC1448756

[B10] Masel J Siegal ML Robustness: mechanisms and consequences Trends Genet 2009 25 9 395 403 10.1016/j.tig.2009.07.005 19717203 PMC2770586

[B11] Milton CC Huynh B Batterham P Rutherford SL AA H Quantitative trait symmetry independent of Hsp90 buffering: Distinct modes of genetic canalization and developmental stability Proc Natl Acad Sci USA 2003 100 23 13396 13401 10.1073/pnas.1835613100 14595030 PMC263825

[B12] Freilich S Anat K Borenstein E Gophna U Sharan R Ruppin E Decoupling environment-dependent and independent genetic robustness across bacterial species PLoS Comp Biol 2010 6 e1000690 10.1371/journal.pcbi.1000690 PMC2829043 20195496

[B13] McGuigan K Sgrò CM Evolutionary consequences of cryptic genetic variation Trends Ecol Evol 2009 24 6 305 311 10.1016/j.tree.2009.02.001 19328588

[B14] Gibson G Dworkin I Uncovering cryptic genetic variation Nat Rev Genet 2004 5 681 690 15372091 10.1038/nrg1426

[B15] Sangster TA Salathia N Undurraga S Milo R Schellenberg K Lindquist S Queitsch C HSP90 affects the expression of genetic variation and developmental stability in quantitative traits Proc Natl Acad Sci USA 2008 105 8 2963 2968 10.1073/pnas.0712200105 18287065 PMC2268568

[B16] Rutherford SL Lindquist S Hsp90 as a capacitor for morphological evolution Nature 1998 396 336 342 10.1038/24550 9845070

[B17] Maisnier-Patin S Roth JR Fredriksson A Nyström T Berg OG Andersson DI Genomic buffering mitigates the effects of deleterious mutations in bacteria Nat Genet 2005 37 1376 1379 10.1038/ng1676 16273106

[B18] Waddington CH Genetic assimialtion of an acquired character Evolution 1953 7 2 118 126 10.2307/2405747

[B19] Wagner GP Evolutionary genetics: the nature of hidden genetic variation unveiled Curr Biol 2003 13 24 R958 R960 10.1016/j.cub.2003.11.042 14680653

[B20] Fares MA Ruiz-González MX Moya A Elena SF Barrio E GroEL buffers against deleterious mutations Nature 2002 417 398 10.1038/417398a 12024205

[B21] Hayden EJ Ferrada E Wagner A Cryptic genetic variation promotes rapid evolutionary adaptation in an RNA enzyme Nature 2011 474 92 97 10.1038/nature10083 21637259

[B22] Sangster TA Salathia N Lee HN Watanabe E Schellenberg K Morneau K Wang H Undurraga S Queitsch C Lindquist S HSP90-buffered genetic variation is common in *Arabidopsis thaliana* Proc Natl Acad Sci USA 2008 105 8 2969 2974 10.1073/pnas.0712210105 18287064 PMC2268569

[B23] Bergman A Siegal M Evolutionary capacitance as a general feature of complex gene networks Nature 2003 10.1038/nature01765 12891357

[B24] Rutherford S Hirate Y Swalla BJ The Hsp90 capacitor, developmental remodeling, and evolution: Therobustness of gene networks and the curious evolvability of metamorphosis Critic Rev Biochem Mol Biol 2007 42 5 355 372 10.1080/10409230701597782 17917872

[B25] Pearl LH Prodromou C Structure and mechanism of the Hsp90 molecular chaperone machinery Ann Rev Biochem 2006 75 1 271 294 10.1146/annurev.biochem.75.103004.142738 16756493

[B26] Yeyati PL Bancewicz RM Maule J van Heyningen V Hsp90 selectively modulates phenotype in vertebrate development PLoS Genet 2007 3 3 e43 10.1371/journal.pgen.0030043 17397257 PMC1839141

[B27] Sgrò CM Wegener B Hoffmann AA A naturally occurring variant of Hsp90 that is associated with decanalization Proc R Soc B: Biol Sci 2010 277 2049 2057 10.1098/rspb.2010.0008 PMC2880099 20200026

[B28] Wagner GP Chiu C-H Hansen TF Is Hsp90 a regulator of evolvability? J Exp Zool (Mol Dev Evol) 1999 285 116 118 10.1002/(SICI)1097-010X(19990815)285:2<116::AID-JEZ3>3.0.CO;2-P 10440722

[B29] Meiklejohn CD Hartl DL A single mode of canalization Trends Ecol Evol 2002 17 10 468 473 10.1016/S0169-5347(02)02596-X

[B30] Gibson G Wagner G Canalization in evolutionary genetics: a stabilizing theory? Bioessays 2000 22 372 380 10.1002/(SICI)1521-1878(200004)22:4<372::AID-BIES7>3.0.CO;2-J 10723034

[B31] van der Straten A Rommel C Dickson B Hafen E The heat shock protein 83 (Hsp83) is required for Raf-mediated signalling in Drosophila The EMBO J 1997 16 1961 1969 10.1093/emboj/16.8.1961 PMC1169799 9155022

[B32] Cutforth T Rubin GM Mutations in hsp83 and c&37 impair signaling by the sevenless receptor tyrosine kinase in Drosophila Cell 1994 77 1027 1036 10.1016/0092-8674(94)90442-1 8020093

[B33] Milton CC Batterham P McKenzie JA Hoffmann AA Effect of E(sev) and Su(Raf) *Hsp83 *mutants and trans-heterozygotes on bristle Ttrait means and variation in *Drosophila melanogaster* Genetics 2005 171 1 119 130 10.1534/genetics.104.038463 16183907 PMC1456505

[B34] Walser J-C Chen B Feder ME Heat-shock promoters: targets for evolution by *P *transposable elements in Drosophila PLoS Genet 2006 2 10 e165 10.1371/journal.pgen.0020165 17029562 PMC1592238

[B35] Chen B Walser JC Rodgers TH Sobota RS Burke MK Rose MR Feder ME Abundant, diverse, and consequential *P *elements segregate in promoters of small heat-shock genes in Drosophila populations J Evol Biol 2007 20 5 2056 2066 10.1111/j.1420-9101.2007.01348.x 17714322

[B36] Lerman DN Feder ME Naturally Occurring Transposable Elements Disrupt hsp70 Promoter Function in Drosophila melanogaster 2005 22 776 783 10.1093/molbev/msi063 15574805

[B37] Chen B Shilova V Zatsepina O Evgenev M ME F Location of *P *element insertions in the proximal promoter region of hsp70A is consequential for gene expression and correlated with fecundity in *Drosophila melanogaster* Cell Stress & Chaperones 2007 13 11 17 18347937 10.1007/s12192-007-0002-4 PMC2666209

[B38] Sabo P Humbert R Hawrylycz M Wallace J Dorschner M Genome-wide identification of DNasel hypersensitive sites using active chromatin sequence libraries Proc Natl Acad Sci USA 2004 101 4537 4542 10.1073/pnas.0400678101 15070753 PMC384782

[B39] Farkas G Leibovitch B Elgin S Chromatin organization and transcriptional control of gene expression in Drosophila Gene 2000 253 117 136 10.1016/S0378-1119(00)00240-7 10940549

[B40] Xiao H Lis JT Heat shock and developmental regulation of the *Drosophila melanogaster *hsp83 gene Mol Cell Biol 1989 9 4 1746 1753 2471067 10.1128/mcb.9.4.1746-1753.1989 PMC362593

[B41] Michaud S Marin R Tanguay RM Regulation of heat shock gene induction and expression during Drosophila development Cell Mol Life Sci 1997 53 1 104 113 10.1007/PL00000572 9117990 PMC11147240

[B42] Rose MR Mueller LD Burke MK Anecdotal, historical and critical commentaries on genetics: new experiments for an undivided genetics Genetics 2011 188 1 10 10.1534/genetics.111.128900 21546546 PMC3120144

[B43] Pemberton JM Wild pedigrees: the way forward Proc R Soc B: Biol Sci 2008 275 1635 613 621 10.1098/rspb.2007.1531 PMC2386891 18211868

[B44] Macdonald SJ Long AD A potential regulatory polymorphism upstream of hairy is not associated with bristle number variation in wild-caught Drosophila Genetics 2004 167 4 2127 2131 10.1534/genetics.104.026732 15342546 PMC1471012

[B45] Khazaeli AA Van Voorhies W Curtsinger JW The relationship between life span and adult body size is highly strain-specific in Drosophila melanogaster Exp Geront 2005 40 5 377 385 10.1016/j.exger.2005.02.004 15919589

[B46] Kristensen TN Loeschcke V Hoffmann AA Can artificially selected phenotypes influence a component of field fitness? Thermal selection and fly performance under thermal extremes Proc R Soc B: Biol Sci 2007 274 1611 771 778 10.1098/rspb.2006.0247 PMC2093976 17251092

[B47] Ometto L Stephan W Lorenzo DD Insertion/deletion and nucleotide polymorphism data reveal constraints in *Drosophila melanogaster *introns and intergenic regions Genetics 2004 169 3 1521 1527 10.1534/genetics.104.037689 PMC1449560 15654088

[B48] Xiao H Lis J Germline transformation used to define key features of heat-shock response elements Science 1988 239 1139 1142 10.1126/science.3125608 3125608

[B49] Sullivan W Ashburner M Hawley RS Cherbas L, Cherbas P Drosophila: A laboratory manual Drosophila cell culture and transformation 2000 20 2 New York: Cold Spring Harbor Laboratory Press

[B50] Sgrò CM Milton CC Jensen LT Frydenberg J Loeschcke V Batterham P Hoffmann AA Nucleotide diversity in the Hsp90 gene in natural populations of *Drosophila melanogaster *from Australia Insect Mol Biol 2008 17 6 685 697 10.1111/j.1365-2583.2008.00843.x 19133078

[B51] Wesley CS WF E Isolation and analysis of the breakpoint sequences of chromosome inversion In(3L)Payne in Drosophila melanogaster Proc Nati Acad Sci USA 1994 91 3132 3136 10.1073/pnas.91.8.3132 PMC43529 8159716

[B52] Charlesworth D Willis JH The genetics of inbreeding depression Nat Rev Genet 2009 10 783 796 10.1038/nrg2664 19834483

[B53] Sangster TA Lindquist S Queitsch C Under cover: causes, effects and implications of Hsp90-mediated genetic capacitance Bioessay 2004 26 348 362 10.1002/bies.20020 15057933

[B54] Michalak P Minkov I Helin A Lerman D Bettencourt B Feder M Korol A Nevo E Genetic evidence for adaptation-driven incipient speciation of Drosophilamelanogaster along a microclimatic contrast in "Evolution Canyon," Israel Proc Nati Acad Sci USA 2001 98 23 13195 13200 10.1073/pnas.231478298 PMC60847 11687637

[B55] Pisa V Cozzolino M Gargiulo S Ottone C Piccioni F Monti M Gigliotti S Talamo F Graziani F Pucci P The molecular chaperone Hsp83 is a component of the cap-binding complex and interacts with the translational repressor Cup during Drosophila oogenesis Gene 2009 432 1-2 67 74 10.1016/j.gene.2008.11.025 19101615

[B56] Yue L Karr TL Nathan DF Swift H Genetic analysis of viable Hsp83 alleles reveals a critical role in Drosophila spermatogenesis Genetics 1999 151 1065 1079 10049923 10.1093/genetics/151.3.1065 PMC1460532

[B57] Ding D Parkhurst SM Halsell SR Lipshitz HD Dynamic Hsp83 RNA localization during Drosophila oogenesis and embryogenesis Mol Cell Biol 1993 13 6 3773 3781 7684502 10.1128/mcb.13.6.3773-3781.1993 PMC359859

[B58] Song Y Fee L Lee TH Wharton RP The molecular chaperone Hsp90 Is required for mRNA localization in *Drosophila melanogaster *embryos Genetics 2007 176 4 2213 2222 10.1534/genetics.107.071472 17565952 PMC1950626

[B59] Haag-Liautard C Dorris M Maside X Macaskill S Halligan DL Charlesworth B Keightley PD Direct estimation of per nucleotide and genomic deleterious mutation rates in Drosophila Nature 2007 445 7123 82 85 10.1038/nature05388 17203060

[B60] Rohmer C David J Moreteau B Joly D Heat induced male sterility in *Drosophila melanogaster*: Adaptive genetic variations among geographic populations and role of the Y chromosome J Exp Biol 2004 207 2735 2743 10.1242/jeb.01087 15235002

[B61] Ayrinhac A Debat V Gibert P Kister AG Legout H al e Cold adaptation in geographical populations of *Drosophila melanogaster*: Phenotypic plasticity is more important than genetic variability Funct Ecol 2004 18 700 706 10.1111/j.0269-8463.2004.00904.x

[B62] Anderson AR Hoffmann AA McKechnie SW Umina PA Weeks AR The latitudinal cline in the In(3R)Payne inversion polymorphism has shifted in the last 20 years in Australian Drosophila melanogaster populations Mol Ecol 2005 14 3 851 858 10.1111/j.1365-294X.2005.02445.x 15723676

[B63] Castrillon DH Gonczy P Alexander S Rawson R Eberhart CG Viswanathan S DiNardo S Wasserman SA Toward a molecular genetic analysis of spermatogenesis in *Drosophila melanogaster*: characterization of male-sterile mutants generated by single P element mutagenesis Genetics 1993 135 2 489 505 8244010 10.1093/genetics/135.2.489 PMC1205651

[B64] Takemori N Yamamoto M-T Proteome mapping of the *Drosophila melanogaster *male reproductive system Proteomics 2009 9 2484 2493 10.1002/pmic.200800795 19343724

[B65] Levene H Contributions to probability and statistics 1960 Palo Alto: Stanford University Press

