# Supplementary material for: Hsp90 is important for fecundity, longevity, and buffering of cryptic deleterious variation in wild fly populations
Source: BMC Evol Biol. 2012 Feb 27;12:25. doi: 10.1186/1471-2148-12-25 (PMC3305614; doi:10.1186/1471-2148-12-25)
Supplement: Additional file 1 — Table S1. The 42 natural populations of D. melanogaster screened for Hsp83 insertion/deletion mutations. Lines F1, F2, F7, and F8 were reared as isofemale lines. The remaining lines were mass-reared. [file 1471-2148-12-25-S1.DOC]

**Additional file 1**

Table S1. The 42 natural populations of *D. melanogaster* screened for *Hsp83* insertion/deletion mutations. The F1, F2, F7, and F8 were reared as isofemale lines, and the other lines mass reared.

| No. | Locality | Country |
| --- | --- | --- |
| F1 | Okayama | Japan |
| F2 | Tokyo | Japan |
| F3 | Taishan | China |
| F4 | Lanzhou | China |
| F5 | Wuwei | China |
| F6 | Kunming | China |
| F7 | Cairns | Australia |
| F8 | Melbourne | Australia |
| F9 | Delhi | India |
| F10 | Mysore | India |
| F11 | Kuala Lumpur | Malaysia |
| F12 | Bordeaux | France |
| F13 | Grande Ferrade | France |
| F14 | Besançon | France |
| F15 | Helsinki | Finland |
| F16 | Seattle | USA |
| F17 | Miami | USA |
| F18 | Hawaii | USA |
| F19 | Oahu, Hawaii | USA |
| F20 | Tahiti | French Polynesia |
| F21 | San Thomas Isd. | Virgin Island |
| F22 | Guyana | French Guiana |
| F23 | Petet Bong | Guadeloupe |
| F24 | Oaxaca | Mexico |
| F25 | Ica | Peru |
| F26 | Montevideo | Uruguay |
| F27 | Seychelles | Seychelles |
| F28 | Marrakech | Morocco |
| F29 | Swaziland | Swaziland |
| F30 | Cotonou | República de Benin |
| F31 | Alexandrie | Egypt |
| F32 | Lamto | Ivory Coast, Côte d'Ivoire |
| F33 | Tai Forest | Ivory Coast, Côte d'Ivoire |
| F34 | Djeffa | Bénin |
| F35 | Port Louis | Mauritius |
| F36 | Rinanga, near Pointe-Noire | Congo |
| F37 | Dimonika | Congo |
| F38 | Madibou | Brazzaville, Congo |
| F39 | Primus-Mai Brazza | Brazzaville, Congo |
| F40 | Tana | Tananarive |
| F41 | Tulear | Madagascar |
| F42 | Marteen | Saint Martin, Caribbean |
