# Supplementary figures and images for: Hsp90 is important for fecundity, longevity, and buffering of cryptic deleterious variation in wild fly populations
Source: BMC Evol Biol. 2012 Feb 27;12:25. doi: 10.1186/1471-2148-12-25 (PMC3305614; doi:10.1186/1471-2148-12-25)

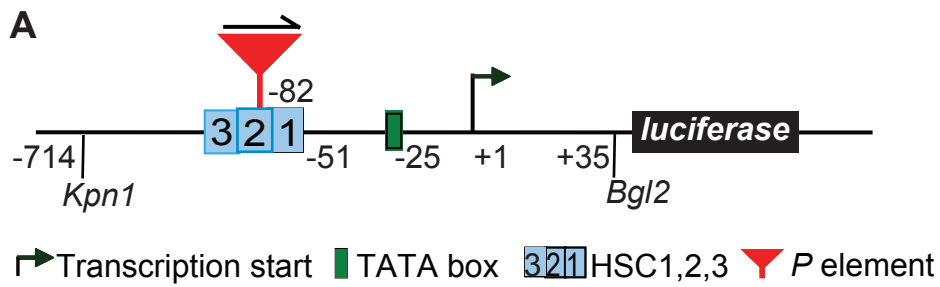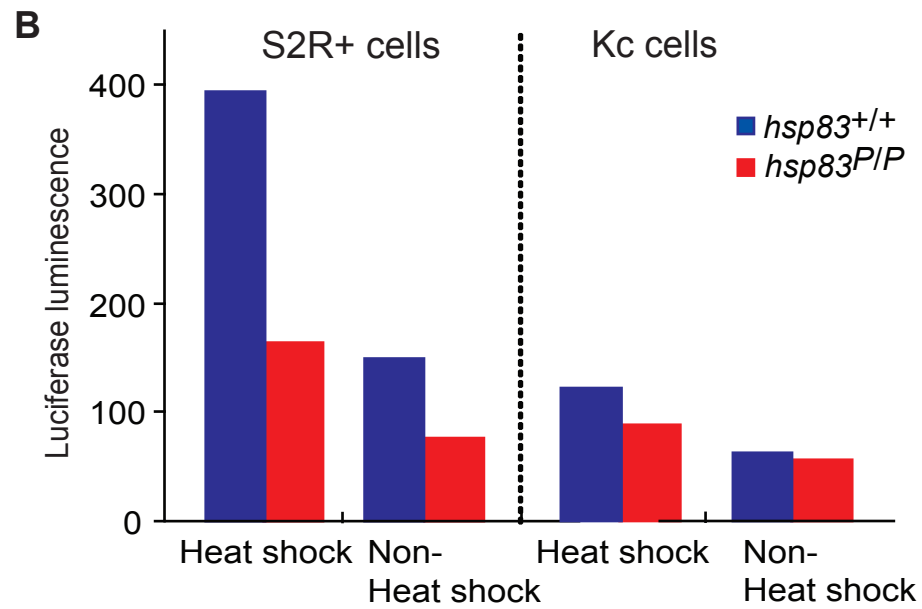

Supplement: Additional file 2 — Figure S1. P element insertion mutation in the Hsp83 proximal promoter reduces Hsp83 gene expression. (A) Hsp83 gene promoter-luciferase reporter constructs (see also Materials and methods). Promoters of the Hsp83 gene (both as wild-type and with P-element insertion) from the Okayama population were amplified, digested with Kpn1 and Bgl2, and ligated into the promoter region of the luciferase gene in the pGL3-basic vector as described in Materials and Methods. HSC, Heat-shock consensus element; TATA, TATA box. Constructs are not drawn to scale. (B) Hsp83 gene promoter activity, as measured by a luciferase assay (see also Materials and methods). Drosophila S2R+ cells and Kc cells were transiently transfected in separate experiments with luciferase constructs that contained the wild-type Hsp83 promoter, and the same promoter with an inserted P element. Transfectants were subject to control (i.e., non-heat-shock) or 37°C heat-shock treatments, as described in Materials and methods. The vertical axis shows the firefly luciferase signal, expressed as the ratio of firefly to Renilla luciferase luminescence measured from 4 replicate cell lines for each treatment. [file 1471-2148-12-25-S2.PDF]

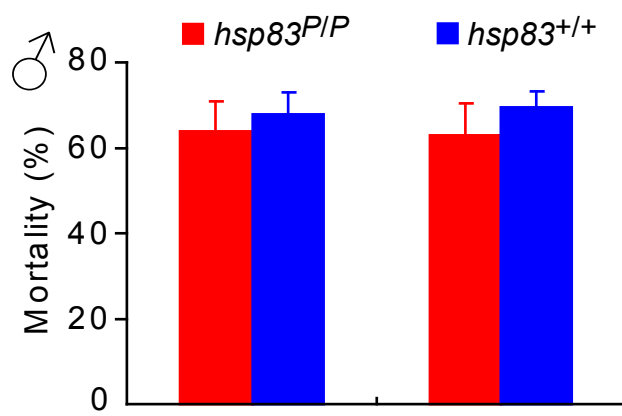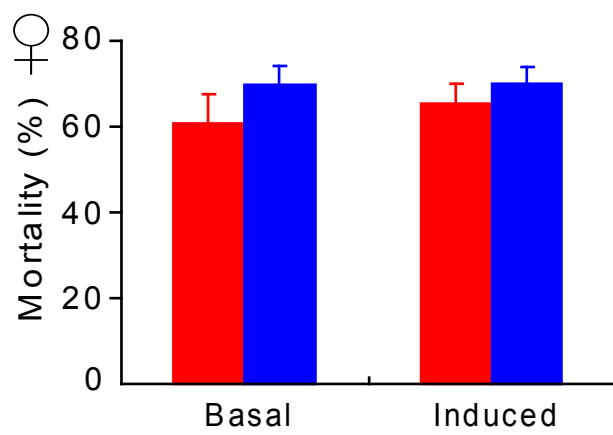

Supplement: Additional file 5 — Figure S2. Mutant flies are no less thermotolerant than wild-type flies. We determined thermal stress tolerance via adult mortality of males (upper panel) and females (lower panel) under heat-shock stress in mutant and wild-type lines. We determined basal thermal tolerance (labeled as 'Basal' in the figure) by heat-shocking 3-day old adults in a water bath at 40.3°C for 30 minutes, and counted survivors after a 24 hour recovery period at 25°C. We measured induced thermal tolerance (labeled as 'Induced') by pre-treating flies at 29.0°C for 20 minutes, followed by 40 minutes at 36.5°C for Hsp83 expression induction, before heat-shocking flies as above at 40.3°C for 30 minutes. We subjected about 30 flies of each genotype (Hsp83P/P and Hsp83+/+) from each of 13 to 20 isofemale lines to this treatment. Error bars indicate one standard error of the mean. [file 1471-2148-12-25-S5.PDF]

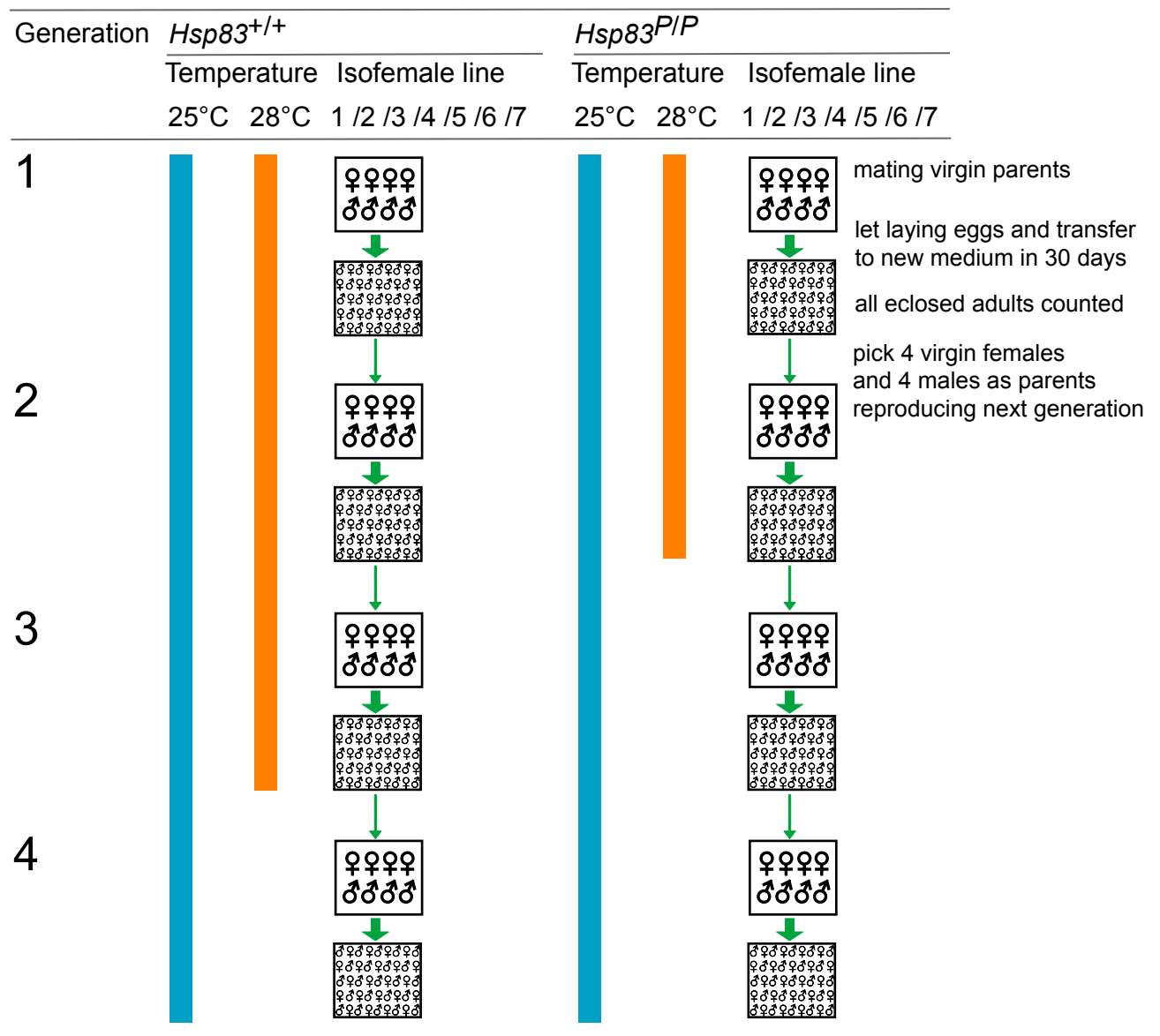

Supplement: Additional file 6 — Figure S3. Experimental design for continuous inbreeding of flies at 25°C and 28°C. Flies from 7 isofemale lines for each genotype (wildtype Hsp83+/+ vs. mutant Hsp83P/P) were subject to the same inbreeding procedure in parallel. In each generation, four 3-day-old virgin females and four 3-day-old males of an isofemale line were mated in a vial to generate the next generation. The 8 flies in the vial were transferred to a new vial with fresh food every other day for a total of 30 days. Adults eclosing from each vial were censused every other day until eclosion ceased. At 25°C inbreeding continued for 4 generations. At 28°C inbreeding continued for 3 generations for wild-type flies, and for 2 generations of mutant flies, because the flies ceased to lay eggs in the second generation. Fly lines isogenized from each population were subject to the same inbreeding procedure. [file 1471-2148-12-25-S6.PDF]

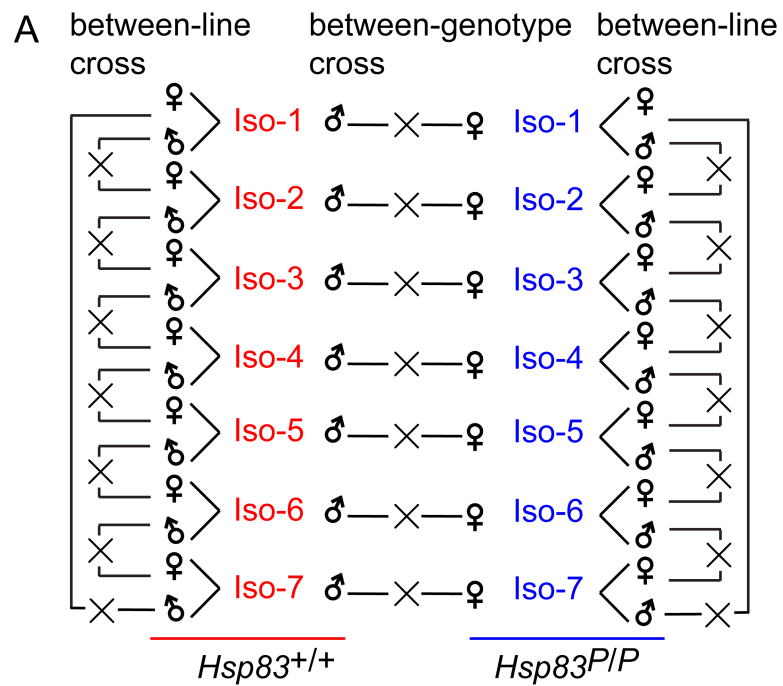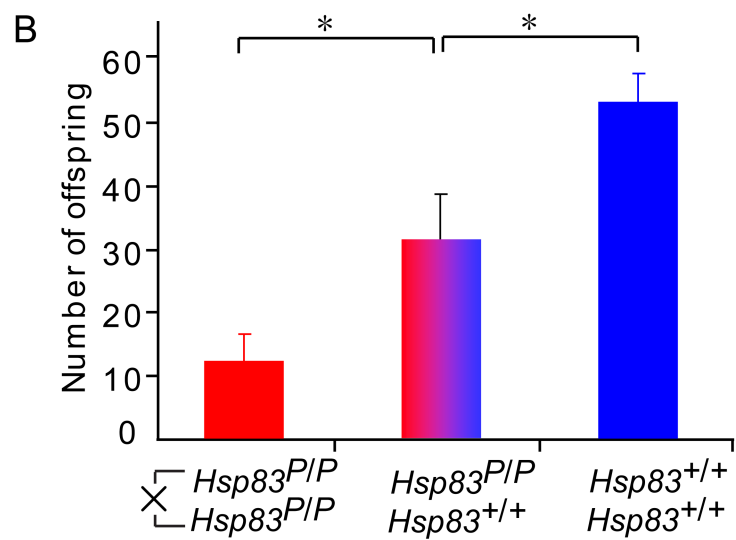

Supplement: Additional file 7 — Figure S4. Outbreeding rescued the fertility loss caused by stress at 28°C in Hsp83 mutants. (A) Experimental design for outbreeding of flies at 28°C. Flies from 7 isofemale lines (iso-1 to iso-7) for each genotype (wildtype Hsp83+/+ vs. mutant Hsp83P/P) were collected from adults eclosed in the second generation of inbreeding at 28°C for the Ivory Coast population (see the inbreeding design in Materials and methods and additional file 6). Virgin females from one line and males from another line with the same genotype were mated to establish between-line crosses. In addition, virgin females from one mutant line and males from a wild-type line were mated to set up between-genotype crosses. In total, seven between-line crosses for each genotype and seven between-genotype crosses were established. In each cross, four 3-day-old females and four 3-day-old males were mated in a vial. The eight flies in the vial were transferred to a new vial with fresh food every other day for 30 days. Adults eclosing from each vial were censused every other day until eclosion ceased. × indicates crossing. (B) female fecundity from crosses between lines and crosses between genotypes. Independent-sample t-tests were performed to calculate significance of fecundity difference between two crosses. Asterisks (*) indicate significant differences at p < 0.05 in these tests. [file 1471-2148-12-25-S7.PDF]

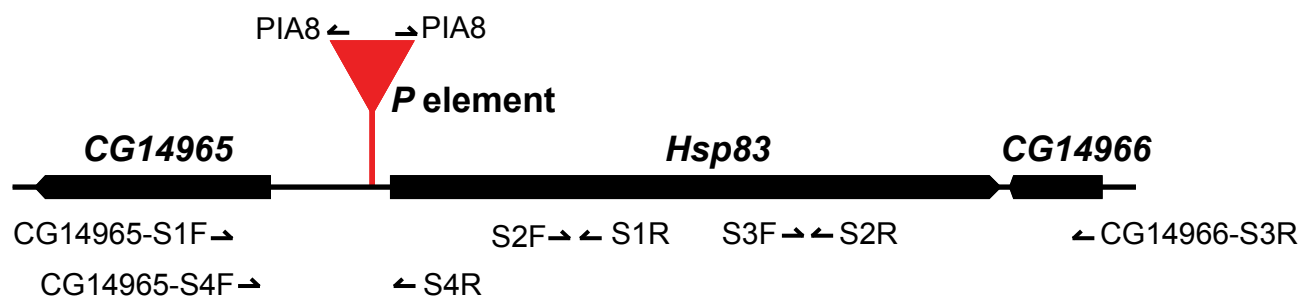

Supplement: Additional file 8 — Figure S5. Position of primers located in the chromosome region containing the genes CG14965, Hsp83, and CG14966. Filled areas correspond to genes (black bars) and to a P element (red triangle). The tapered ends of black bars indicate the direction in which a gene is transcribed. Primers are described in Materials and methods. The arrow near each primer indicates the primer's 5' > 3' direction. Primer sequences are shown in additional file 1. The sequence is drawn to scale. [file 1471-2148-12-25-S8.PDF]
