# Supplementary material for: Hsp90 is important for fecundity, longevity, and buffering of cryptic deleterious variation in wild fly populations
Source: BMC Evol Biol. 2012 Feb 27;12:25. doi: 10.1186/1471-2148-12-25 (PMC3305614; doi:10.1186/1471-2148-12-25)
Supplement: Additional file 3 — Table S2. Frequency of Hsp83P/P and Hsp83P/+ alleles occurring in three populations. [file 1471-2148-12-25-S3.DOC]

**Additional file 3**

Table S2. Frequency of *Hsp83^P^*/*^P^* and *Hsp83^P^*^/+^ alleles occurring in three populations. *N* is the number of flies subjected to frequency determination.

| Origin of mutant allele | *Hsp83^P^*/*^P^* frequency | *Hsp83^P^*^/+^ frequency | Departure from Hardy-Weinberg disequilibrium (χ^2^ test) |
| --- | --- | --- | --- |
| Okayama (*N*=312) | 0.3% | 3.2% | *p*=0.037 |
| Tokyo (*N*=55) | 0 | 5.5% | *p*=0.978 |
| Ivory Coast (*N*=39) | 0 | 15.4% | *p*=0.593 |
