# Supplementary material for: Hsp90 is important for fecundity, longevity, and buffering of cryptic deleterious variation in wild fly populations
Source: BMC Evol Biol. 2012 Feb 27;12:25. doi: 10.1186/1471-2148-12-25 (PMC3305614; doi:10.1186/1471-2148-12-25)
Supplement: Additional file 4 — Table S3. No association between frequency of the inversion Inv(3L)P and Hsp83 alleles. [file 1471-2148-12-25-S4.DOC]

**Additional file 4**

Table S3. No association between frequency of the inversion *Inv(3L)P* and Hsp83 alleles detected.

|  | *Hsp83* mutant | |  | *Hsp83* wild-type | |  | Fisher’s exact test |
| --- | --- | --- | --- | --- | --- | --- | --- |
|  | Standard | *Inv(3L)P* |  | Standard | *Inv(3L)P* |  |  |
| Okayama | 13 | 1 |  | 11 | 0 |  | *p*=1.00 |
| Ivory Coast | 11 | 1 |  | 15 | 0 |  | *p*=0.44 |
