# Supplementary material for: Hsp90 is important for fecundity, longevity, and buffering of cryptic deleterious variation in wild fly populations
Source: BMC Evol Biol. 2012 Feb 27;12:25. doi: 10.1186/1471-2148-12-25 (PMC3305614; doi:10.1186/1471-2148-12-25)
Supplement: Additional file 9 — Table S4. Sequences of the primers used in Materials and Methods. [file 1471-2148-12-25-S9.DOC]

**Additional file 9**

Table S4. Sequences of the primers used in Materials and methods

| Primer name | Sequence (5’>3’) | Notes |
| --- | --- | --- |
| CG14965-S1F | TTAggTgTTgCTgCTTTggAg | The three primer pairs (CG14965-S1F and S1R, S2F and S2R, S3F and CG14966-S3R) were used to amplify 3 overlapping regions covering *Hsp83*, the 5’ part of its upstream gene CG14965, and the downstream gene CG14966. |
| S1R | CATCgTTgTTCTTggAggTgA |
| S2F | GTCCGACCTGGTCAACAACT |
| S2R | GGACTTCATCAGCTTGCACA |
| S3F | GACAAGGCCAAGTTCGAGAG |
| CG14966-S3R | ACGCCCAAATAATGTCGAAG |
| CG14965-S4F | GAAGAGCTTCACCGTTCTGG | To amplify the intergenic region between *Hsp83* and the CG14965 gene located immediately upstream *Hsp83* |
| S4R | GACGCTGCTTGTTGTTACGA |
| PIA8 | CgggACCACCTTATgTTAT | *P* element specific primer complementary to the inverted repeat flanking the *P* element |
| *Hsp83*F | GTGCAAGCTGATGAAGTCCA | To amplify *Hsp83* cDNA and produce a 465bp fragment in quantitative PCR |
| *Hsp83*R | GAAGCGTCCTCTGTGTCCTC |
| actin88F1F | ACCGGTATTGTGCTGGA | To amplify the actin88F gene to produce a 216bp fragment for endogenous control in quantitative PCR |
| actin88F1R | AGCCACGTAGCACAGCTTCT |
| Kpn1F1 | GAGGTACC GGGAGGCATCGATAACAGAA | To amplify a 749bp fragment of the wild-type promoter to construct a luciferase reporter plasmid, restriction sites for Kpn1 and Bgl2 are indicated by underlined type |
| Bgl2R1 | CGAGATCT AAACTTTTCAGACGCTGCTTG |
