# Supplementary material for: Hsp90 is important for fecundity, longevity, and buffering of cryptic deleterious variation in wild fly populations
Source: BMC Evol Biol. 2012 Feb 27;12:25. doi: 10.1186/1471-2148-12-25 (PMC3305614; doi:10.1186/1471-2148-12-25)
Supplement: Additional file 10 — Table S5. Fly populations and isofemale lines used for trait measurements. [file 1471-2148-12-25-S10.DOC]

**Additional file 10**

Table S5. Fly populations and isofemale lines used for trait measurements

| Traits | Population | Isofemale lines | Methods for generating replicated genotypes |
| --- | --- | --- | --- |
| Competition assay | Okayama  Tokyo  Ivory Coast | One mass reared isofemale line from each population | First assay: 1:1 ratio of the two genotypes for each population;  Second assay: different proportions of mutant and wild-type flies from Okayama. |
| fecundity | Okayama | Five isofemale lines homozygous for *Hsp83^P/P^* and five for *Hsp83*^+/+^ | Five females with five males were mated for each line. |
| longevity |  | Five isofemale lines homozygous for *Hsp83*^+/+^ and five for *Hsp83^P^*/*^P^* | Forty flies of the same sex and same age from each of the ten lines were examined for each line. |
| thermal tolerance |  | Thirteen to twenty isofemale lines | Samples of about 30 flies for each line were treated. |
| Inbreeding effects | Okayama  Tokyo  Ivory Coast | Seven isogenic lines for genotype *Hsp83*^+/+^ and for *Hsp83^P^*/*^P^* | Four 3-day-old virgin females and four 3-day-old males of a line were mated for each line. |
| Outbreeding effects | Ivory Coast | Seven isofemale lines homozygous for *Hsp83*^+/+^ and for *Hsp83^P^*/*^P^* | Seven crosses were established within each genotype and between genotype, respectively. In each cross, four 3-day-old females and four 3-day-old males were mated. |
